# Medium‐Dose Formoterol Attenuated Abdominal Aortic Aneurysm Induced by EPO via β2AR/cAMP/SIRT1 Pathway

**DOI:** 10.1002/advs.202306232

**Published:** 2024-02-14

**Authors:** Jianlin Zhang, Yu Cao, Ruiqing Ren, Wenhai Sui, Yun Zhang, Meng Zhang, Cheng Zhang

**Affiliations:** ^1^ National Key Laboratory for Innovation and Transformation of Luobing Theory The Key Laboratory of Cardiovascular Remodeling and Function Research Chinese Ministry of Education Chinese National Health Commission and Chinese Academy of Medical Sciences Department of Cardiology Qilu Hospital of Shandong University Jinan Shandong 250012 China; ^2^ Cardiovascular Disease Research Center of Shandong First Medical University Central Hospital Affiliated to Shandong First Medical University Jinan 250013 China

**Keywords:** abdominal aortic aneurysm, bioinformatics, erythropoietin, formoterol, senescence

## Abstract

Abdominal aortic aneurysm (AAA) is a life‐threatening vascular disease but effective drugs for treatment of AAA are still lacking. Recently, erythropoietin (EPO) is reported to induce AAA formation in apolipoprotein‐E knock out (ApoE^−/−^) mice but an effective antagonist is unknown. In this study, formoterol, a β2 adrenergic receptor (β2AR) agonist, is found to be a promising agent for inhibiting AAA. To test this hypothesis, ApoE^−/−^ mice are treated with vehicle, EPO, and EPO plus low‐, medium‐, and high‐dose formoterol, respectively. The incidence of AAA is 0, 55%, 35%,10%, and 55% in these 5 groups, respectively. Mechanistically, senescence of vascular smooth muscle cell (VSMC) is increased by EPO while decreased by medium‐dose formoterol both in vivo and in vitro, manifested by the altered expression of senescence biomarkers including phosphorylation of H2AX^serine139^, senescence‐associated β‐galactosidase activity, and P21 protein level. In addition, expression of sirtuin 1 (SIRT1) in aorta is decreased in EPO‐induced AAA but remarkably elevated by medium‐dose formoterol. Knockdown of β2AR and blockage of cyclic adenosine monophosphate (cAMP) attenuate the inhibitory role of formoterol in EPO‐induced VSMC senescence. In summary, medium‐dose formoterol attenuates EPO‐induced AAA via β2AR/cAMP/SIRT1 pathways, which provides a promising medication for the treatment of AAA.

## Graphical abstract

Formoterol binds to β2AR and activates cAMP, which increases SIRT1 protein expression, leading to suppressed VSMC senescence induced by EPO. In contrast, SIRT1 is downregulated by EPO via CBL activation, resulting in aggravated VSMC senescence. Thus, medium‐dose formoterol attenuated EPO‐induced AAA via β2AR/cAMP/SIRT1 pathways, which provides a promising medication for the treatment of AAA.

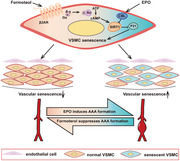

## 1 Introduction

Abdominal aortic aneurysm (AAA) is a potentially fatal vascular disorder commonly seen in elderly men and is usually defined by an increased diameter of the abdominal aorta reaching 3.0 cm or more.^[^ ^1^ ^]^ Patients with AAA may remain asymptomatic for decades until the onset of aneurysmal rupture with a high mortality rate exceeding 80%.^[^ ^2^ ^]^ As an animal model mimicking human AAA is lacking and the mechanism of AAA is unclear, no pharmacological agent has been proven effective for inhibiting AAA development.^[^ ^3^ ^]^ Among several risk factors contributing to the formation and development of AAA, aging is a major one, with 8% of men aged > 65 years suffering from AAA, and this incidence increases by 40% every five years of age in this population.^[^ ^4^ ^]^ Age‐related alterations, such as inflammation, vascular stiffening, and oxidative stress, make the vessel more susceptible to AAA.^[^ ^5^, ^6^, ^7^ ^]^ Accumulating evidence shows that vascular smooth muscle cell (VSMC) senescence plays a crucial role in vascular aging. For instance, increased telomere attrition and DNA double‐strand breaks were found in cultured VSMCs from human AAA.^[^ ^8^ ^]^ Age‐related reduction of Sirtuin 1 (SIRT1) in VSMCs accelerates vascular aging and promotes the formation and rupture of AAA.^[^ ^4^ ^]^ These findings highlight the importance of VSMC senescence in AAA.

Formoterol, a β2 adrenergic agonist, is widely used for symptomatic relief in asthma and control of chronic obstructive pulmonary disease (COPD).^[^ ^9^, ^10^ ^]^ The therapeutic mechanism of formoterol involves both bronchodilation through airway smooth muscle relaxation and anti‐inflammatory activity.^[^ ^10^ ^]^ Intracellular signaling following β2 adrenergic receptor (β2AR) activation is mainly dependent on a trimeric heterotrimeric G stimulatory protein coupled to adenylate cyclase that increases cellular cyclic adenosine monophosphate (cAMP) synthesis,^[^ ^9^, ^11^ ^]^ which induces airway relaxation through phosphorylation of muscle regulatory proteins and attenuation of cellular Ca^2+^ concentrations.^[^ ^11^ ^]^ In addition, accumulating evidence suggested the potential role of cAMP signaling in AAA. As an example, our previous study demonstrated that smooth muscle‐specific knockout of the α subunit of the heterotrimeric G stimulatory protein (Gsα) reduced cAMP production and exaggerated AAA in mice.^[^ ^12^ ^]^ Inhibition of both cAMP production and SIRT1 activation promoted VSMC senescence and aggravated the development of AAA.^[^ ^13^ ^]^ However, the effect of formoterol on AAA has not been reported.

Erythropoietin (EPO) is traditionally deemed an essential mediator of red blood cell production, but recent studies indicate that the role of EPO extends beyond erythropoiesis. Pathological processes including inflammation, infections, and autoimmunity, have been found to be related to EPO.^[^ ^14^ ^]^ The classical EPO‐inducible pathways include the Janus kinase 2 (JAK2) and signal transducer and activator of the transcription 5 (STAT5) signal pathway.^[^ ^15^ ^]^ In parallel to the JAK2‐STAT5 pathway, EPO is also associated with pathways inducing inflammatory cytokine production.^[^ ^16^ ^]^ In 2021, our group reported a novel mouse model of AAA induced by EPO injection in both wild type and apolipoprotein E knock out (ApoE^−/−^) mice,^[^ ^17^ ^]^ with many pathological features resembling human AAA but without noticeable effect on blood pressure, serum lipid profile, and liver and renal function.^[^ ^17^ ^]^ Mechanistically, we found EPO triggered AAA through its homodimer receptors, EPOR, and induced angiogenesis mainly via JAK2‐STAT5 in endothelium cells.^[^ ^17^ ^]^ Besides, inflammatory response, collagen degradation, and VSMC apoptosis also played important roles in EPO‐induced AAA.^[^ ^17^ ^]^ However, the mechanism underlying EPO‐induced AAA is not completely understood and an effective medication for suppressing the progression of EPO‐induced AAA is yet to be developed.

In the present study, we explored the main pathways underlying EPO‐induced AAA by means of bioinformatic analysis, and then by using the connectivity map (CMap) database, we discovered medium‐dose formoterol as an effective medication to suppress the formation of EPO‐induced AAA in ApoE^−/−^ mice. Mechanistically, we found that VSMC senescence was significantly increased in aortas receiving EPO injection, but substantially alleviated by medium‐dose formoterol treatment via activated β2AR‐cAMP‐SIRT1 pathway.

## 2 Results

### 2.1 Identification and Analysis of DEGs in GSE174556 Dataset

**Figure 1:**
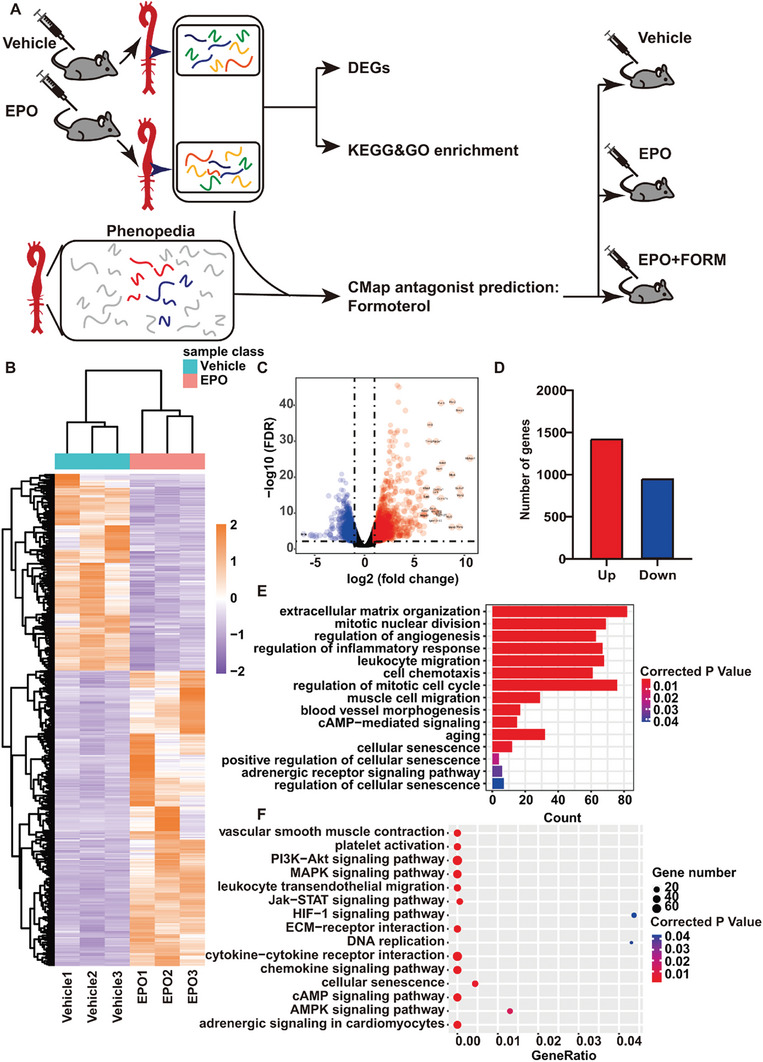
Analysis of differential expression genes in GSE174556 dataset. A) Flow chart shows our bioinformatic and experimental strategy. GSE174556 dataset was composed of the RNA‐seq data of entire aortas isolated from ApoE^−/−^ mice receiving EPO (*n = 3*) and vehicle (*n = 3*) treatment. We conducted differential expression genes (DEGs) and GO&KEGG enrichment analyses on the dataset. AAA‐centered dataset provided by Phenopedia intersected with GSE174556 dataset. Formoterol was chosen by CMap as the compound to confront EPO‐induced‐AAA in later experiments. We testified the effect of formoterol by injection of formoterol to ApoE^−/−^ mice in EPO‐induced AAA model. B) Heatmap of all DEGs in GSE174556 dataset resulting from a two‐way hierarchical clustering. C) Volcano map of DEGs. The blue dots represent the downregulated DEGs and the red ones represent the upregulated DEGs. D) The number of significantly upregulated and downregulated genes. E) Gene ontology (GO) enrichment for DEGs in GSE174556 dataset. F) Kyoto Encyclopedia of genes and genomes (KEGG) enrichment for DEGs in GSE174556 dataset.

The mRNA expression profiling dataset GSE174556 was acquired from the Gene Expression Omnibus (GEO) database, which contained mRNA expression data in the aortic tissues of three mice with EPO‐induced AAA and three mice with normal aortas.^[^ ^17^ ^]^ **Figure** **1**A provides an overview of our bioinformatic and experimental strategy. The heatmap of total DEGs distinguished the two groups from each other clearly (Figure 1B). With the use of the Volcano Plot and “edgeR” package of R, 2,382 DEGs were identified, of which 1,430 genes were upregulated and 952 genes were downregulated (Figure 1C,D).

To further investigate the biological behaviors of these DEGs, we performed Gene Ontology (GO) (Figure 1E) and Kyoto Encyclopedia of Genes and Genomes (KEGG) (Figure 1F) enrichment analysis. According to GO enrichment analysis, mitotic nuclear division and regulation of the mitotic cell cycle were significantly enriched, suggesting that biological processes related to the cell cycle may play an essential role in EPO‐induced AAA. Cellular senescence is characterized by cell cycle arrest in the G1 or G2 phase.^[^ ^18^ ^]^ Moreover, both GO and KEGG enrichment analysis indicated that cellular senescence and cAMP signaling pathways were significantly related to EPO‐induced AAA.

**Figure 2:**
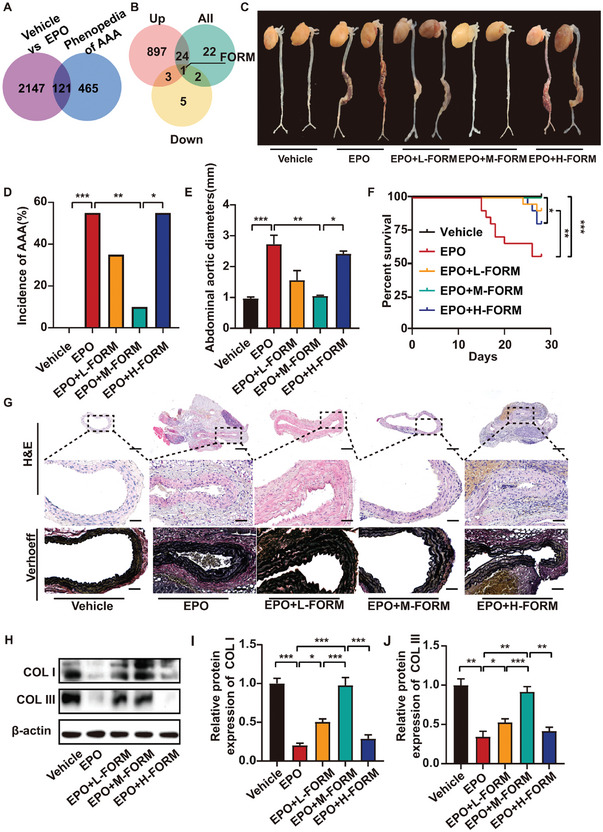
Formoterol (FORM) suppressed the formation of EPO‐induced AAA in ApoE^−/−^ mice. A) Venn diagram showing the intersection of DEGs and AAA‐centered gene set provided by Phenopedia B) Venn diagram showing that formoterol was the only option (number “1” in Figure 2B) in the intersection among Connectivity map results based on all DEGs, upregulated DEGs and downregulated DEGs. C) Representative photographs of the abdominal aortic specimens in ApoE^−/−^ mice receiving vehicle, EPO, EPO+low‐dose formoterol, EPO+medium‐dose formoterol, EPO+high‐dose formoterol treatment, respectively. D)The incidence of AAA in each group (*n =* 20 per group). E) Abdominal aorta diameters in each group (*n =* 8 per group). F) The survival rate in each group (*n =* 20 per group). G) Representative H&E staining and Verhoeff staining of abdominal aortic section in ApoE^−/−^ mice receiving vehicle, EPO, EPO+low‐dose formoterol, EPO+medium‐dose formoterol, EPO+high‐dose formoterol treatment, respectively. Low (×4, scale bars = 100 µm), and medium (×20, scale bars = 25 µm), magnifications are shown. H) Representative western blot analysis of collagen I (COL I) and collagen III (COL III) expression in abdominal aortas of 5 groups of mice. I‐J) Quantitative analysis of COL I and COL III expression in abdominal aortas of 5 groups of mice (*n =* 6 per group). Fisher's exact test was used for (D). The Kruskal‐Wallis test followed by Nemenyi post hoc test was used for (E). Log‐rank (Mantel‐Cox) test was used for (F). The other data were analyzed via One‐way ANOVA followed by the Tukey test for post hoc comparison. *p < 0.05, **p <0.01, ***p <0.001, mean ± SEM.

Phenopedia is a database providing a disease‐associated view of genetic association studies. It summarizes information about genes studied in relation to particular diseases.^[^ ^19^ ^]^ We intersected the differential genes from the GSE174556 dataset and AAA‐associated genes obtained from Phenopedia and found 121 genes in the intersection (**Figure** **2**A). We then established the gene co‐expression network and protein‐protein interaction network of the intersection genes to explore the crucial genes in the intersection. Genes related to collagen degradation and inflammation were in the crucial positions of the network (Figure S1, Supporting Information), indicating that extracellular matrix homeostasis and inflammation played important roles in EPO‐induced AAA.

The Connectivity Map (CMap), an online platform to compare input gene signatures with gene patterns in the contexts of over 450 000 chemical compounds, is a tool to identify compounds that create gene expression patterns opposite to or strengthening specific gene expression differences with a view to repurposing old drugs to new application.^[^ ^20^, ^21^, ^22^, ^23^ ^]^ With the aim of finding molecules that might prevent and reverse EPO‐induced AAA, we uploaded the intersection genes to CMap. Formoterol was the only candidate in the intersection of the three sets, including candidates that could reverse global gene alteration, candidates that might inhibit the expression of upregulated genes, and candidates that might promote the expression of downregulated genes (Figure 2B). Formoterol, a common anti‐asthmatic β2‐agonist previously reported to suppress airway inflammation and dilate bronchus, was predicted to be capable of creating gene expression patterns against gene expression changes induced by EPO, and thus, might reverse the effects of EPO on the aorta. Additionally, pathways strongly associated with formoterol, including VSMC contraction and adrenergic receptor signaling pathway, were enriched from DEGs of GSE174556 dataset (Figure 1E,F). Therefore, we identified formoterol as a potential compound to suppress EPO‐induced AAA.

### 2.2 Formoterol Dose‐Dependently Affected EPO‐Induced AAA

In the first in vivo experiment, we successfully replicated the EPO‐induced AAA model in ApoE^−/−^ mice. A schematic diagram showing the experiment procedure of ApoE^−/−^ mice were given in Figure S2 (Supporting Information). Similar to our previous report,^[^ ^17^ ^]^ the incidence of AAA was 55% and the mortality was 45% due to AAA rupture in the EPO group (Figure 2C–F). Moreover, EPO injection did not affect body weight, heart rate, blood pressure as well as serum lipid profiles of mice relative to the vehicle group (Figure S3, Supporting Information), which was in agreement with our previous results.^[^ ^17^ ^]^ As formoterol was suggested by CMap database that might alleviate the EPO effect on aortas, we examined the dose‐response relation between formoterol and EPO‐induced AAA. The doses of 0.3 mg kg^−1^ day^−1^ and 1 mg kg^−1^ day^−1^ of formoterol were chosen because these doses were proven to improve recovery in spinal cord and renal injuries.^[^ ^24^, ^25^ ^]^ There was no significant difference in body weight, mean and diastolic blood pressure, and serum lipid profiles in three formoterol‐treated groups versus EPO‐ or vehicle‐treated groups. However, high‐dose formoterol significantly increased heart rate and systolic blood pressure compared with other groups (Figure S3, Supporting Information). Representative photographs of aortic specimens in the five groups of ApoE^−/−^ mice were shown in Figure 2C. The incidence of AAA was 0%, 55%, 35%, 10%, and 55% in the vehicle group, EPO group, EPO+low‐dose formoterol group, EPO+medium‐dose formoterol group, and EPO+high‐dose formoterol group, respectively (Figure 2D). These findings indicated that the effect of formoterol on EPO‐induced AAA was dose‐dependent and U‐shaped. Low‐ and medium‐dose formoterol was protective against AAA whereas high‐dose formoterol lost such efficacy. Moreover, the diameters of aortas exhibited a similar trend in three EPO+formoterol groups compared with the EPO group (Figure 2E). Similarly, the survival rate was the lowest in the EPO group, which was significantly increased in the EPO+low‐dose formoterol and EPO+medium‐dose formoterol groups. In contrast, the survival rate was decreased again when the dose of formoterol increased from the medium to the high level (Figure 2F), again exhibiting a dose‐dependent and U‐shaped effect. Morphology of all aortic specimens in 5 groups of mice and representative pathologic staining of the aortas from mice who died of AAA rupture were exhibited in Figure S4A,B (Supporting Information). H&E and Verhoeff staining revealed that both EPO and EPO+high‐dose formoterol treatment caused aortic wall thickening and disruption of elastic fibers in the aorta, which was virtually reversed by EPO+medium‐dose formoterol treatment and only slightly improved by EPO+low‐dose formoterol treatment (Figure 2G). Taken together, medium dose formoterol was effective in preventing AAA formation in EPO‐induced AAA model in ApoE^−/−^ mice.

In the second in vivo experiment, to explore the effect of formoterol treatment alone on the formation of AAA without EPO pretreatment, we performed intraperitoneal injections of saline, low‐dose of formoterol, medium‐dose of formoterol, and high‐dose formoterol into 4 groups of ApoE^−/−^ mice, respectively. We found that in comparison with the vehicle group of mice, formoterol treatment alone exerted no effect on the morphology and pathology of aortas regardless of the doses of formoterol used (Figure S5, Supporting Information). These results have several important implications: first, β2AR agonism with formoterol had no effect on the morphology of mouse aorta; second, the beneficial effects of medium‐dose formoterol on AAA came into play only in the presence of EPO stimulation; and third, the null effect of high‐dose formoterol on AAA development cannot be explained by the direct harmful effect of high‐dose formoterol on mouse aortic tissues.

### 2.3 Formoterol Modulated the Expression and Activity of Extracellular Matrix‐Related Proteins In Vivo

**Figure 3:**
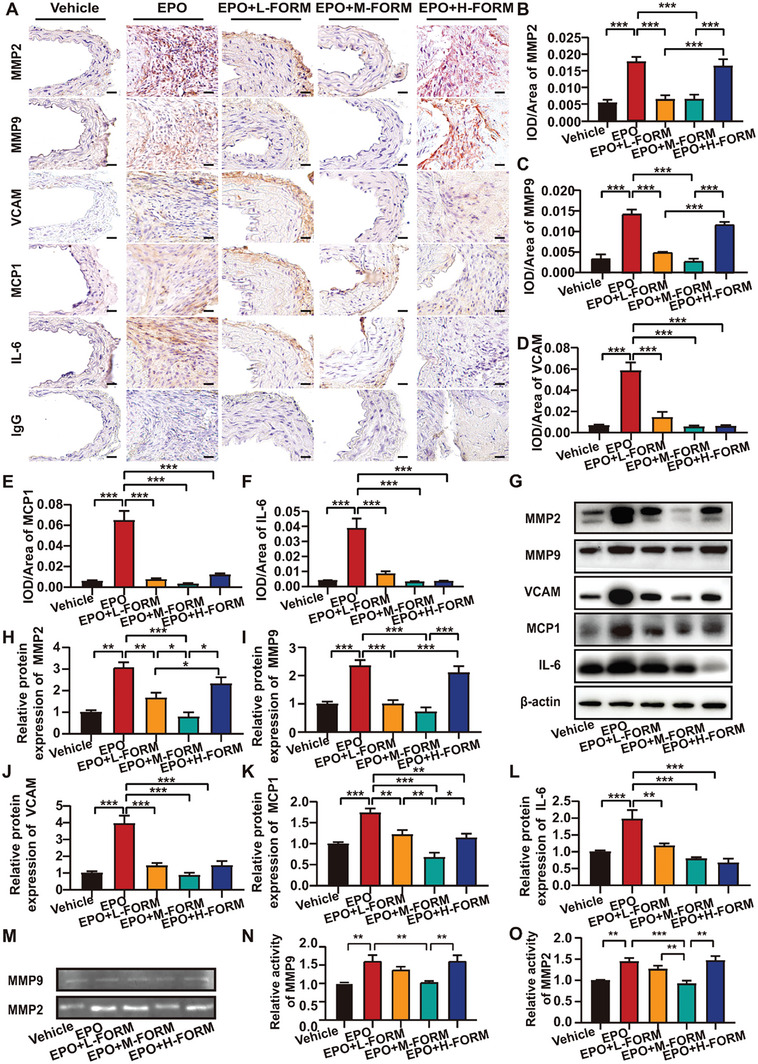
Effect of formoterol (FORM) on expression of matrix metalloproteinases (MMPs) and proinflammatory cytokines in EPO‐induced AAA. A) Representative immunohistochemical staining of the abdominal aortic sections for MMP2, MMP9, VCAM, MCP1, and IL‐6 in 5 groups of mice (scale bars = 25 µm) B‐F) Quantitative analysis of MMP2, MMP9, VCAM, MCP1 and IL‐6 expression in 5 groups of mice (*n =* 6 per group). G) Representative western blot analysis of MMP2, MMP9, VCAM, MCP1, and IL‐6 expression in abdominal aortas of 5 groups of mice and their quantitative analysis H‐L) (*n =* 6 per group). M) Representative zymography of MMP2 and MMP9 activity in abdominal aortas of 5 groups of mice and their quantitative analyses (N‐O) (*n =* 6 per group). *p <0.05, **p <0.01, ***p <0.001, One‐way ANOVA followed by Tukey test for post hoc comparison, mean ± SEM.

To examine the effect of formoterol on extracellular matrix homeostasis, we determined the expression of type I and type III collagen and matrix metalloproteinase (MMP)2 and MMP9 in the aortic wall in mice. Western blot showed that the protein expression of type I and type III collagen in the aortic wall was substantially decreased in the EPO group compared with the vehicle group, which was partially reversed in EPO + low‐dose formoterol group and almost completely reversed in EPO + medium‐dose formoterol group compared with EPO group. In contrast, the expression of type I and type III collagen in the aortic wall again declined in EPO + high‐dose formoterol group relative to the EPO + medium‐dose formoterol group (Figure 2H–J). Both immunohistochemical staining and western blot showed that protein expression of MMP2 and MMP9 in aortic tissues was significantly increased in EPO group versus the vehicle group, which returned to the baseline level in EPO + low‐dose formoterol and EPO + medium‐dose formoterol groups while increased again in EPO + high‐dose formoterol group (Figure 3A–C,G–I). The activity of MMP2 and MMP9 showed similar changes in the five groups of mice (Figure 3M–O). Thus, low‐dose and medium‐dose formoterol treatment reversed EPO‐induced extra matrix degradation.

### 2.4 Formoterol Inhibited the Expression of Proinflammatory Cytokines In Vivo

In the first in vivo experiment, we further examined the expression of proinflammatory cytokines, including interleukin 6 (IL‐6), vascular cell adhesion molecule (VCAM), and monocyte chemoattractant protein 1 (MCP1), in the aortas of 5 groups of mice by both immunostaining and western blot analysis. The results showed that protein expression level of IL‐6, MCP1, and VCAM in aortic tissues was significantly increased in EPO‐induced AAA compared with the vehicle group, which was attenuated by low‐, medium‐ and high‐dose formoterol treatment relative to the EPO group (Figure 3A,D–G,J–L). Thus, formoterol treatment, regardless of doses used, attenuated EPO‐induced inflammation in the aortic tissues in vivo.

### 2.5 Effect of Formoterol on Cell Apoptosis in Aortic Tissues

**Figure 4:**
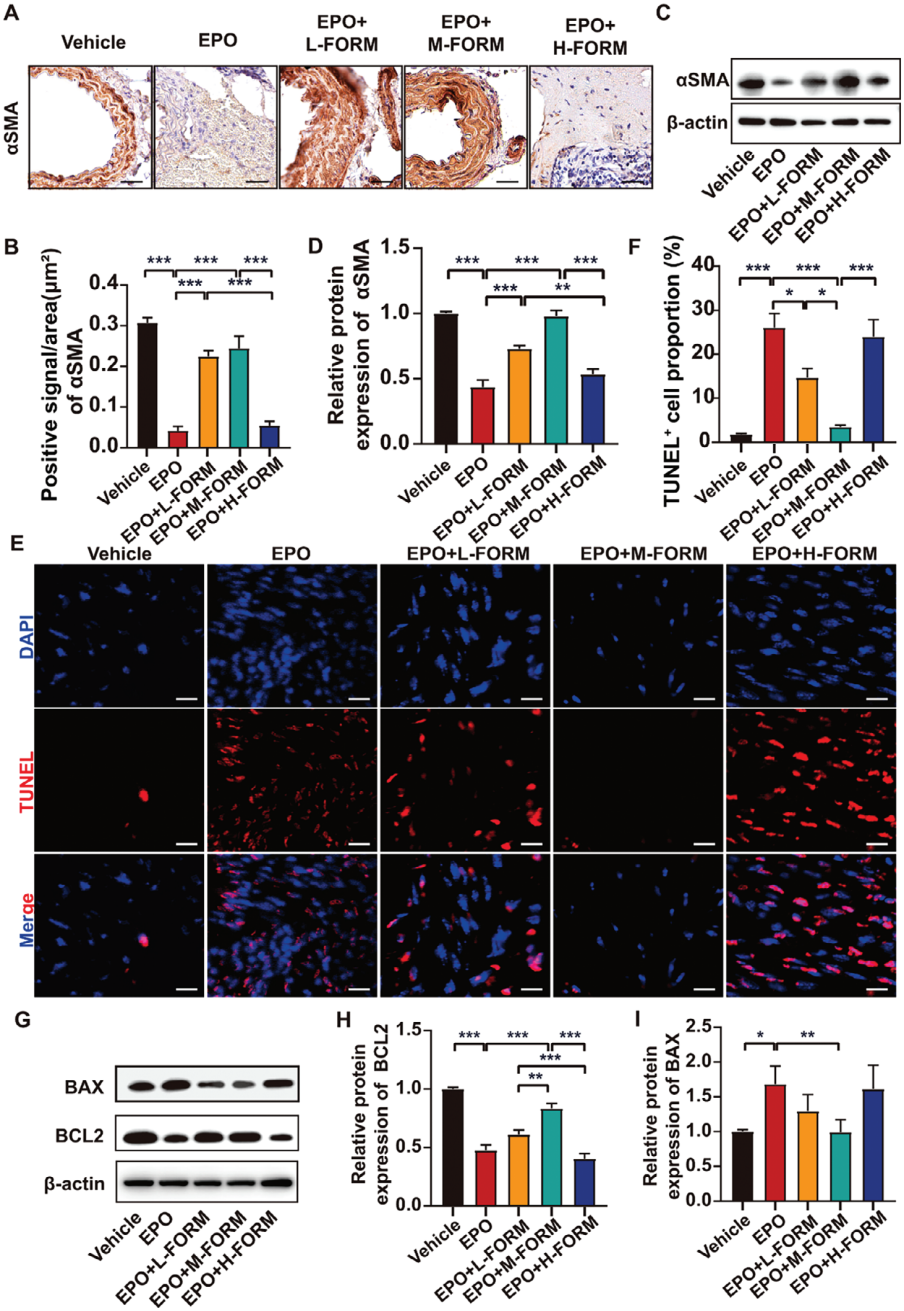
Effect of formoterol (FORM) on EPO‐induced apoptosis of abdominal aortic tissues. A) Representative immunohistochemical staining of the abdominal aortic sections for αSMA in 5 groups of mice (scale bars = 10 µm) B) Quantitative analysis of αSMA expression in the abdominal aortic sections of 5 groups of mice (*n =* 6 per group) C) Representative western blot analysis of αSMA expression in abdominal aortas of 5 groups of mice and quantitative analysis (D). E) Representative TUNEL images in abdominal aortas of 5 groups of mice (scale bars = 25 µm) and quantitative analysis (F). G) Representative western blot analysis of BAX and BCL2 in abdominal aortas of 5 groups of mice and quantitative analysis (H) and (I) (*n =* 6 per group). *p <0.05, **p <0.01, ***p <0.001, One‐way ANOVA followed by Tukey test for post hoc comparison, mean ± SEM.

As the reduced content of VSMCs in aortic tissues is a striking pathological feature of AAA,^[^ ^26^ ^]^ we then measured the expression of α‐smooth muscle actin (αSMA), a marker of SMCs, in the abdominal aortic wall of mice by both immunohistochemical staining and western blot analysis. The results showed that the expression of αSMA in the AAA from the EPO group was markedly lower than that in the vehicle group (**Figure** **4**A–D). Treatment with low‐ and medium‐dose formoterol exerted a beneficial effect on αSMA expression of the content of VSMCs, while treatment with high‐dose formoterol abolished these beneficial effects (Figure 4A–D).

To assess the effect of formoterol on cell apoptosis in EPO‐induced AAA, TUNEL assays were performed, which showed that EPO treatment significantly increased apoptosis, whereas low‐ and medium‐dose formoterol markedly attenuated apoptosis induced by EPO. However, high‐dose formoterol reversed these salutary effects (Figure 4E,F). We also measured the expression levels of Bax and Bcl2 in the abdominal aortic tissues (Figure 4G–I), which showed that Bcl2 expression was significantly down‐regulated in the EPO group but up‐regulated in the medium‐dose formoterol group. High‐dose formoterol treatment again reversed the protective effect of medium‐dose formoterol treatment (Figure 4G,H). Compared with the vehicle group, EPO injection markedly increased the expression of Bax which was attenuated by medium‐dose formoterol treatment (Figure 4G–I). Altogether, these results indicated that low‐ and medium‐dose formoterol treatment alleviated apoptosis of VSMCs in the aortic tissues induced by EPO while high‐dose formoterol abolished the protective effect of formoterol on EPO‐induced AAA.

### 2.6 Medium‐Dose Formoterol Mitigated Aortic VSMC Senescence in EPO‐induced AAA

**Figure 5:**
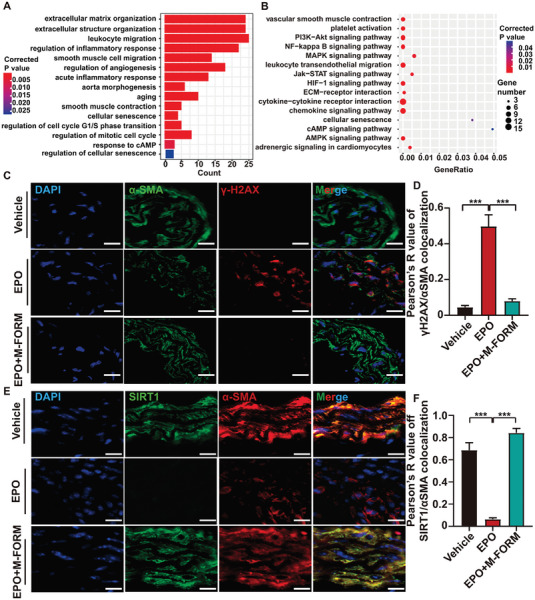
Formoterol (FORM) suppressed EPO‐induced VSMC senescence in mouse aorta. A) GO enrichment for the intersection genes. B) KEGG enrichment for the intersection genes. C) Representative immunofluorescent analysis of the abdominal aortic sections for detecting the colocalization (yellow particles) of γH2AX (red particles) and αSMA (specific for SMCs, green particles) in ApoE^−/−^ mice receiving vehicle, EPO and EPO+ medium‐dose formoterol treatment, respectively (scale bars = 10 µm). D) Quantitative analysis of γH2AX/αSMC colocalization in (C) (*n =* 6 per group). E) Representative immunofluorescent analysis of the abdominal aortic sections for detecting the colocalization (yellow particles) of SIRT1 (green particles) and αSMA (specific for SMCs, red particles) in ApoE^−/−^ mice receiving vehicle, EPO and EPO+medium‐dose formoterol treatment, respectively. (scale bars = 10 µm). F) Quantitative analysis of γH2AX/αSMC colocalization in (E) (*n =* 6 per group). *p <0.05, **p <0.01, ***p <0.001, One‐way ANOVA followed by Tukey test for post hoc comparison, mean ± SEM.

Previous studies have reported that VSMC senescence is related to AAA formation, consistent with the clinical finding that aging is the major risk factor for AAA.^[^ ^4^ ^]^ Moreover, analysis of both GSE174556 dataset (Figure 1E,F) and the 121 intersection genes (**Figure** **5**A,B) indicated that cell cycle pathways and senescence might be involved in EPO‐induced AAA. And one of the senescence biomarkers was phosphorylation of Histon H2AX at serine139, known as γH2AX, which derived from DNA double‐strand breaks. In an attempt to reveal the role of senescence in both EPO‐induced AAA and the effect of formoterol on EPO‐induced AAA, we examined the number of γH2AX foci in the aortic tissues in vehicle, EPO, and EPO + medium‐dose formoterol groups. Immunofluorescent staining of γH2AX and αSMA in aortas revealed increased γH2AX foci in VSMCs of EPO‐induced AAA relative to the vehicle group, and γH2AX foci were scanty in VSMCs of EPO + medium‐dose formoterol group (Figure 5C,D).

SIRT1 belongs to a family of nicotinamide adenine dinucleotide (NAD^+^)‐dependent deacetylases with extraordinary abilities to prevent diseases and even reverse the aging process.^[^ ^27^ ^]^ Highly expressed SIRT1 has been proven to protect vessels from VSMC senescence and AAA.^[^ ^4^ ^]^ By Immunofluorescent analysis, we found SIRT1 expression was significantly down‐regulated in VSMCs of EPO‐induced AAA compared with that in the vehicle group, which were up‐regulated in VSMCs of EPO + medium‐dose formoterol group relative to EPO group (Figure 5E,F). Taken together, in the aortic tissues, the senescent level was higher in the EPO‐induced AAA group and was attenuated in the EPO + medium‐dose formoterol group.

### 2.7 Formoterol Ameliorated Aortic Senescence via β2AR

**Figure 6:**
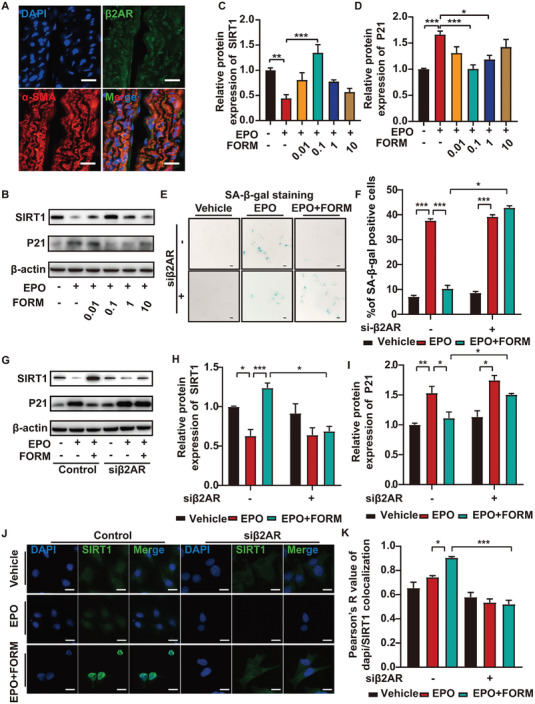
Formoterol ameliorated aortic senescence via β2AR. A) Representative immunofluorescent analysis of the abdominal aortic sections for detecting the colocalization (yellow particles) of β2AR (green particles) and αSMA (specific for SMCs, red particles) in the aorta of ApoE^−/−^ mice (scale bars = 10 µm). B) Representative western blot analysis of protein expression of SIRT1 and P21 in VSMCs treated with vehicle, EPO, EPO+0.01 nmol mL^−1^ formoterol, EPO+0.1 nmol mL^−1^ formoterol, EPO+1 nmol mL^−1^ formoterol and EPO+10 nmol mL^−1^ formoterol, respectively. C,D) Quantitative analysis of western blot in (B) (*n =* 6 per group). E) Representative sections of SA‐β‐gal staining of VSMCs transfected with β2AR siRNA (siβ2AR) or negative control siRNA (siNC) and treated with vehicle, EPO and EPO+0.1 nmol mL^−1^ formoterol, respectively. (scale bars = 10 µm). F) Quantitative analysis of percentage of VSMCs with positive SA‐β‐gal staining in (E) (*n =* 6 per group). G) Representative western blot analysis of protein expression of SIRT1, P21 in VSMCs transfected with β2AR siβ2AR or siNC and treated with vehicle, EPO and EPO + 0.1 nmol mL^−1^ formoterol, respectively. H‐I) Quantitative analysis of western blot in (G) (*n =* 6 per group). J) Representative images of immunostaining of SIRT1 in VSMCs transfected with β2AR siβ2AR or siNC and treated with vehicle, EPO, and EPO+ 0.1 nmol mL^−1^ formoterol, respectively. K) Quantitative analysis of colocalization of dapi/SIRT1 in VSMCs in(G) (*n =* 6 per group). *p < 0.05, **p <0.01, ***p <0.001, Two‐way ANOVA followed by Tukey test for post hoc comparison, mean ± SEM.

β2AR belongs to the β‐adrenergic receptor family containing three members, β1AR, β2AR, and β3AR, which share 51% sequence identity and play an important role in many physiological and pathological processes.^[^ ^28^, ^29^ ^]^ It is well known that both selective and non‐selective β‐blockers affect cardiac and vascular function.^[^ ^30^ ^]^ β2AR is mostly expressed in smooth muscle cells in the body. Formoterol, an inhaled long‐acting β2‐agonist, has been used as a bronchodilator combating asthma and COPD for decades.^[^ ^31^, ^32^ ^]^ To explore the mechanisms underlying formoterol protection in EPO‐induced AAA, we examined the expression of β2AR in mouse VSMCs. The double staining of β2AR and αSMA in the normal aortas of mice (**Figure** **6**A) showed that β2AR staining was observed mainly in VSMCs in the aortas.

The expression of P21 protein, a senescence biomarker, was assessed in vitro. P21 is an inhibitor of cyclin‐dependent kinase that blocks cellular proliferation in response to DNA damage and thus accumulates in senescent cells.^[^ ^13^ ^]^ To explore the dose‐response effect of formoterol in cultured VSMCs, we used a series of concentrations of formoterol in the experiment in vitro. 5 IU mL^−1^ EPO was widely recommended in the literature and used in our early study.^[^ ^17^ ^]^ We found that cultured VSMCs exposed to EPO exhibited lower SIRT1 expression and higher P21 expression levels compared to the vehicle group. In contrast, SIRT1 expression was significantly upregulated by 0.1 nmol mL^−1^ formoterol in addition to EPO treatment compared with that in the EPO group. 0.1 nmol mL^−1^ and 1 nmol mL^−1^ formoterol in addition to EPO treatment suppressed P21 expression relative to EPO treatment alone (Figure 6B–D). Considering the effects on both SIRT1 and P21 levels, 0.1 nmol mL^−1^ formoterol was chosen for the latter experiments. In addition, β2AR internalization was elicited by 10 nmol mL^−1^ formoterol treatment only in cultured VSMC, while treatment with lower doses of 0.01 nmol mL^−1^, 0.1 nmol mL^−1^ and 1 nmol mL^−1^ of formoterol didn't induce β2AR internalization (Figure S7A,B, Supporting Information). Western blot of membrane protein expression of VSMC demonstrated a similar trend in Figure S7C,D (Supporting Information).

As formoterol was a β2‐agonist, we suspected that the effect of formoterol on VSMC senescence was mediated via β2AR. To test this hypothesis, small interfering RNA of β2AR (siβ2AR) and negative control siRNA (siNC) were introduced to cultured VSMCs. Widely used in the detection of senescence, senescence‐associated β‐galactosidase (SA‐β‐gal) activity is a marker of enhanced lysosomal content in aging cells. The results of SA‐β‐gal staining indicated that EPO‐induced VSMC senescence and the addition of formoterol significantly attenuated senescent levels of VSMCs exposed to EPO. Moreover, transfection of siβ2AR blocked the protection of formoterol against EPO‐induced VSMC senescence (Figure 6E,F). Accordingly, in EPO+0.1 nmol mL^−1^ formoterol group, the expression of SIRT1 in VSMCs was significantly reduced by siβ2AR transfection compared with siNC transfection. The reduction of P21 expression level by formoterol treatment was blocked by siβ2AR transfection (Figure 6G–I). Moreover, the immunostaining of SIRT1 in cultured VSMCs showed that the addition of 0.1 nmol mL^−1^ formoterol significantly increased the expression of SIRT1 in the nucleus compared with the EPO group. In contrast, nuclear translocation of SIRT1 induced by formoterol was inhibited by β2AR interference (Figure 6J,K). As previous studies suggested, phosphorylation of SIRT1 leads to nuclear localization and enzymatic activation, thereby promoting cellular survival.^[^ ^33^ ^]^ In contrast, the knockdown of SIRT1 significantly promoted VSMC senescence and P21 expression in both EPO treatment and EPO+formoterol treatment groups (Figure S8A‐D, Supporting Information). These results indicated an essential role of β2AR in the protection of formoterol from EPO‐induced VSMC senescence and upregulation of SIRT1 by formoterol.

### 2.8 cAMP Regulates Formoterol Suppression of Senescence

**Figure 7:**
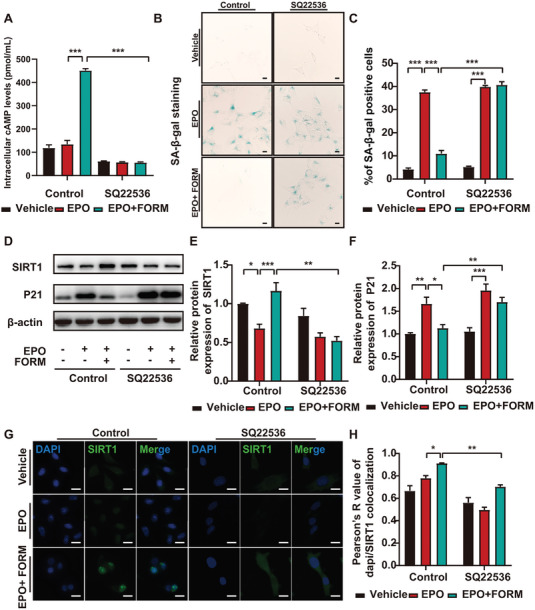
cAMP‐regulated the effect of formoterol on VSMC senescence. A) Quantification of intracellular cAMP levels in VSMC treated with mAC inhibitor SQ22536 (80 µM) or control solution DMSO receiving vehicle, EPO and EPO+0.1 nmol mL^−1^ formoterol, respectively. B) Representative images of SA‐β‐gal staining of VSMC treated with mAC inhibitor SQ22536 (80 µM) or control solution DMSO receiving vehicle, EPO and EPO+0.1 nmol mL^−1^ formoterol, respectively. (scale bars = 10 µm). C) Quantitative analysis of SA‐β‐gal staining in (B) (*n =* 6 per group). D) Representative western blot assay of SIRT1, P21 in VSMC treated with mAC inhibitor SQ22536 (80 µM) or control solution DMSO receiving vehicle, EPO and EPO+0.1 nmol mL^−1^ formoterol, respectively. E‐F) Quantitative analysis of protein expression of SIRT1, P21 protein levels by western blot in (D), (*n =* 6 per group). G) Representative images of immunostaining of SIRT1 in VSMC treated with mAC inhibitor SQ22536 (80 µM) or control solution DMSO receiving vehicle, EPO and EPO+0.1 nmol mL^−1^ formoterol, respectively. H) Quantitative analysis of colocalization of dapi/SIRT1 in (G) (*n =* 6 per group). *p < 0.05, **p <0.01, ***p <0.001, Two‐way ANOVA followed by Tukey test for post hoc comparison, mean ± SEM.

It was reported that activation of β2AR increases the cellular cAMP synthesis and that β2AR/cAMP exerts anti‐inflammatory effects on the respiratory system.^[^ ^9^ ^]^ cAMP has also been found to bind directly to and activate SIRT1.^[^ ^9^, ^13^ ^]^ In this study, cAMP‐related pathway was also enriched in GO and KEGG analysis of DEGs in GSE174556 dataset (Figure 1E,F) and the intersection genes (Figure 5A,B). To determine whether cAMP participates in the formoterol repression of senescence in EPO‐induced AAA, we blocked cAMP synthesis with an inhibitor of membrane adenylyl cyclase (mAC), SQ22536. We found that formoterol significantly upregulated the intracellular cAMP levels in cultured VSMC which was blocked by SQ22536 treatment (**Figure** **7**A). SA‐β‐gal activity assay showed that inhibition of mAC was associated with increased positive SA‐β‐gal staining regardless of the addition of EPO or EPO+formoterol (Figure 7B,C). Western blot analysis suggested that inhibition of mAC diminished SIRT1 upregulation by formoterol and alleviated P21 suppression by formoterol (Figure 7D–F). Immunostaining of SIRT1 also showed that nuclear expression of SIRT1 induced by formoterol was blocked by SQ22536 (Figure 7G,H). These results suggest that the inhibitory effect of formoterol on senescence was reversed by SQ22536, implying that cAMP produced by mAC was vital for formoterol‐mediated regulation of senescence.

### 2.9 Casitas B‐lineage lymphoma (CBL) was Essential for EPO‐induced SIRT1 Reduction

**Figure 8:**
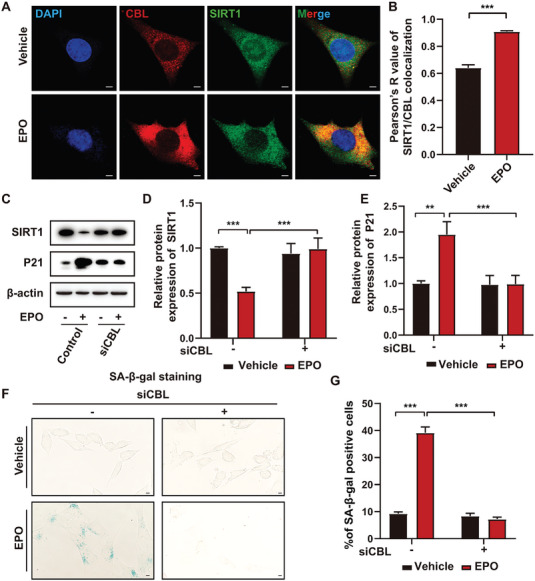
Casitas B‐lineage lymphoma (CBL) was essential for EPO‐induced VSMC senescence. A) Representative immunofluorescent analysis of the colocalization (yellow particles) of CBL (red particles) and SIRT1 (green particles) in VSMC receiving vehicle and EPO treatment respectively (scale bars = 10 µm). B) Quantitative analysis of CBL/SIRT1 colocalization in (A) (*n =* 6 per group). C) Representative western blot analysis of protein expression of SIRT1 and P21 in VSMC transfected with siCBL or negative control siRNA (siNC) and treated with vehicle and EPO respectively. D‐E) Quantitative analysis of western blot in (C) (*n =* 6 per group). F) Representative sections of SA‐β‐gal staining of VSMC transfected with CBL siRNA (siCBL) or negative control siRNA (siNC) and treated with vehicle and EPO respectively (scale bars = 10 µm). G) Quantitative analysis of the percentage of VSMC with positive SA‐β‐gal staining in (F) (*n =* 6 per group). **p <0.01, ***p <0.001, unpaired two‐tailed Student's t‐tests with Welch's correction were applied in (B). The other data were analyzed via Two‐way ANOVA followed by the Tukey test for post hoc comparison, mean ± SEM.

CBL is an E3 ubiquitin‐protein ligase that can be activated by EPO^[^ ^34^ ^]^ and once activated, CBL transfers ubiquitin to substrates promoting degradation of the substrates by the proteasome.^[^ ^35^ ^]^ Furthermore, it has been revealed that CBL combines with SIRT1, triggering K48‐linked polyubiquitination of SIRT1 and leading to SIRT1 degradation.^[^ ^36^ ^]^ Here, we found that colocalization of SIRT1 and CBL increased upon EPO treatment in cultured VSMC (**Figure** **8**A,B). Furthermore, to explore the role of CBL in EPO‐induced VSMC senescence, siCBL and siNC were introduced to cultured VSMC. We found that the downregulation of SIRT1 and upregulation of P21 induced by EPO treatment in vitro were attenuated by CBL knockdown (Figure 8C–E). The results of SA‐β‐gal staining showed that the knockdown of CBL significantly suppressed EPO‐induced VSMC senescence (Figure 8F,G).

## 3 Discussion

There were several important findings in the present study. First, 121 genes were involved in the formation of AAA induced by EPO; Second, formoterol was predicted to be able to create gene expression patterns opposite to effects of EPO on aortas; Third, formoterol exhibited a dose‐dependent and U‐shaped effect on EPO‐induced AAA, and formoterol at a medium dose remarkedly reduced the incidence of EPO‐induced AAA, dilation of abdominal aortas and mortality of mice with AAA; Fourth, medium‐dose formoterol attenuated VSMC senescence induced by EPO via β2AR and subsequent activation of cAMP. To the best of our knowledge, our study was the first to report the beneficial effects and underlying mechanisms of formoterol on EPO‐induced AAA in the literature.

Angiotensin II(AngII)‐induced AAA is a classical animal model widely used in AAA research. There are fundamental differences between EPO‐induced AAA and AngII‐induced AAA. First, in AngII‐induced AAA, hyperlipidemia is essential for AAA formation, and mice with genetic defects in lipid metabolism such as ApoE^−/−^ mice and Ldlr^−/−^ mice are required for this model. By comparison, in EPO‐induced AAA, wild‐type mice without hyperlipidemia may manifest AAA upon EPO treatment, which is in line with clinical observations that hyperlipidemia is only a weak risk factor for patients with AAA.^[^ ^17^, ^33^ ^]^ Second, elevated blood pressure is consequential in AngII‐induced AAA where infusion of a very high dose of Ang II is mandatory, while EPO treatment exerts no effect on blood pressure in mice. Third, in EPO‐induced AAA, angiogenesis and inflammation originating from endothelial cells play a key role, which elicits collagen degradation and VSMC apoptosis in the aortas, whereas in AngII‐induced AAA, the cell target of Ang II is VSMC.^[^ ^17^ ^]^

AAA is a potentially lethal vascular disease with no effective pharmacological therapy^[23]^. Formoterol as a long‐acting, inhaled β2‐agonist not only controls asthma attacks but also improves symptoms and pulmonary function.^[^ ^37^ ^]^ In a mouse model of acute lung injury, the use of formoterol reduced the systemic expression of IL‐6 and improved cardiovascular dysfunction.^[^ ^38^ ^]^ It has been found that patients with asthma tended to be more susceptible to AAA and aortic rupture.^[^ ^39^ ^]^ Furthermore, in mice with allergic lung inflammation, AngII infusion led to doubled abdominal aortic diameter, more intensive macrophage, and mast cell infiltration, loss of arterial media smooth muscle cells, and angiogenesis in AAA lesions.^[^ ^40^ ^]^ Here, we have discovered a dose‐dependent and U‐shaped effect of formoterol in EPO‐induced AAA, and medium‐dose formoterol dramatically reduced the incidence and mortality of AAA in ApoE−/− mice after EPO injection, suggesting that formoterol may be considered as a potential drug for clinical treatment of patients with AAA.

Although the pathogenetic mechanism of AAA has been extensively studied in several animal models, the mechanism underlying the therapeutic effects of formoterol on EPO‐induced AAA is unexplored. Previous studies have reported that formoterol exhibits anti‐inflammatory effects and reduces systemic expression of IL‐6,^[^ ^31^ ^]^ which is well‐known for its contribution to cardiovascular dysfunction. Our results showed that formoterol dose independently inhibited expression of IL‐6, MCP1, and VCAM in EPO‐induced AAA, which is consistent with the anti‐inflammatory effects of formoterol in respiratory diseases, suggesting that anti‐inflammation may be the key mechanism of formoterol treatment in EPO‐induced AAA. In addition, we found that low‐ and medium‐dose formoterol treatment alleviated apoptosis of VSMCs in the aortic tissues induced by EPO, suggesting anti‐apoptosis is another important therapeutic mechanism of formoterol in EPO‐induced AAA. However, as revealed by our previous study,^[^ ^17^ ^]^ EPO treatment per se did not induce VSMC apoptosis in vitro, while treatment with medium from EPO‐pretreated endothelial cells increased VSMC apoptosis and decreased VSMC proliferation, which suggested that EPO‐induced VSMC apoptosis is via endothelial cells. Whether other forms of cell death such as ferroptosis may also play a role in EPO‐induced VSMC reduction remains to be clarified.

Although previous studies found a vital role of renin angiotensin aldosterone system (RAAS) activation in the pathogenesis of AAA and a close relation between β1AR and RAAS,^[^ ^41^, ^42^ ^]^ β1AR is highly expressed in the heart but weakly expressed in VSMCs, as demonstrated by previous studies.^[^ ^43^ ^]^ In the present study, high‐dose formoterol treatment resulted in a higher systolic pressure and heart rate in mice, suggesting a stimulating effect of formoterol on β1AR in the mouse heart, However, these effects may not be used to explain the adverse effect of high‐dose formoterol on AAA. Thus, it is unlikely that RAAS is involved in the therapeutic mechanism of formoterol in EPO‐induced AAA.

AAA is regarded as an age‐related disease because the incidence of AAA increases by 40% every 5 years in men aged > 65 years.^[^ ^4^ ^]^ Cultured VSMCs from human AAA exhibit increased telomere attrition, DNA double‐strand breaks, and limited lifespan^[^ ^13^ ^]^. SIRT1 plays a vital role in metabolism and lifespan improvement,^[^ ^27^ ^]^ and previous studies have reported that SIRT1 prevents stress‐associated vascular remodeling, vascular stiffness and dissection, and atherosclerosis in mice, indicating the importance of SIRT1 for vascular health.^[^ ^4^ ^]^ The inhibitory effect of SIRT1 in VSMCs has been confirmed in AngII‐ and CaCl_2_‐induced AAA models.^[^ ^4^ ^]^ In this study, we demonstrated that EPO treatment downregulated whereas medium‐dose formoterol treatment upregulated SIRT1 expression in vivo and in vitro. Our findings of VSMC senescence in EPO‐induced AAA are consistent with our previous report of vascular inflammation and collagen degradation by EPO injection in mice.^[^ ^17^ ^]^ Moreover, we found that medium‐dose formoterol upregulated SIRT1 expression and protected mouse aortas from AAA, which agrees with a previous report that overexpression of SIRT1 in VSMCs of ApoE^−/−^ mice suppressed VSMC senescence and AAA formation.^[^ ^4^ ^]^ Taken together, medium‐dose formoterol may reverse the aging process in EPO‐induced AAA via SIRT1 upregulation.

In the current study, we found that formoterol attenuated VSMC senescence via β2AR activation. There were few studies of β‐AR signaling in AAA in the literature. Several animal studies have indicated that propranolol, a β‐blocker, may be beneficial to AAA because of both hemodynamic properties and biochemical effects on matrix proteins.^[^ ^44^ ^]^ However, in clinical trials, there was a very small, non‐significant protective effect of propranolol on AAA expansion.^[^ ^45^ ^]^ In the present study, we discovered the therapeutic effects of formoterol, a β2‐agonist, in EPO‐induced AAA, and gene knockdown of β2AR in VSMCs promoted cellular senescence and inhibited SIRT1 expression. These findings suggest that activation of β2AR signaling may be protective against AAA and β2‐agonists might serve as a new therapeutic avenue to the medical treatment of AAA.

One possibility exists that EPO may bind and interfere with β2AR to reduce intracellular cAMP levels. EPOR and cytokine receptor common subunitβ (CD131) are the two receptors that need to form a dimer to bind EPO,^[^ ^46^ ^]^ and these receptors are structurally different from the monomeric receptor β2AR. In addition, the structure difference between EPO and ligands of β2AR, epinephrine, and norepinephrine, are enormous. EPO is a 30‐kDa protein comprising 165 amino acids^[^ ^46^ ^]^ while epinephrine and norepinephrine are small molecules with the chemical formula of C9H13NO3 and C8H11NO3, respectively. Furthermore, we measured the intracellular cAMP levels of cultured VSMC receiving vehicle and EPO treatment. EPO treatment did not suppress the cAMP levels compared with vehicle treatment. Thus, it is unlikely that EPO binds β2AR directly and suppresses cAMP levels.

Upon activation, signal transduction of β2AR occurs via adenylate cyclase, which upregulates the concentration of intracellular cAMP.^[^ ^9^ ^]^ Increasing evidence indicates that cAMP signaling plays an important role in VSMC phenotype switch and cellular senescence in AAA.^[^ ^12^, ^13^ ^]^ Our group previously reported that smooth muscle‐specific knockout of Gsα induced a smooth muscle phenotype switch from a contractile to a synthetic state and exaggerated AngII‐induced AAA.^[^ ^12^ ^]^ Mechanistic studies further showed that cAMP stimulated SIRT1 activation, possibly through a direct interaction leading to subsequent SIRT1 level increasement.^[^ ^13^ ^]^ In support of this finding, our results showed that formoterol upregulated SIRT1 expression and alleviated VSMC senescence through β2AR activation followed by increased expression of cAMP. These results connected the classical cAMP pathway in formoterol‐induced β2AR activation with SIRT1 upregulation and VSMC senescence alleviation, inspiring future drug discovery along the β2AR‐cAMP pathway for the medical treatment of AAA.

A critical question raised by this study is why the rewarding effect of medium‐dose‐formoterol in EPO‐induced AAA was abolished by high‐dose formoterol administration. Because β1AR is minimally expressed in VSMC, non‐selective stimulation of β1AR by high dose‐formoterol, as manifested by increased heart rate and systolic blood pressure in this group of mice, may not explain the null effect of high dose‐formoterol on EPO‐induced AAA. Our results showed that collagen I and III expression were decreased in the aortas of the EPO+high‐dose formoterol group, whereas both expression and activity of MMPs in the aortic tissues were remarkedly increased in EPO+high‐dose formoterol group relative to EPO+low‐dose and EPO+medium‐dose formoterol groups. In addition, the reduction and apoptosis of VSMC were more significant in the EPO+high‐dose formoterol group than in the EPO+low‐dose and EPO+medium‐dose formoterol groups. Nonetheless, compared with the EPO group, the EPO+high‐dose formoterol group showed similar pathological, cellular, and molecular changes in the AAA tissues, indicating a loss‐of‐function effect of high‐dose formoterol on AAA. It has been well known that β2AR desensitization occurs when β2AR is exposed to excessive agonists.^[^ ^47^ ^]^ The mechanism involves the uncoupling of β2AR from adenylate cyclase and the internalization of β2AR.^[^ ^11^ ^]^ In this study, the null effect of high‐dose formoterol on EPO‐induced AAA may be related to β2AR internalization. To test this hypothesis, we performed in vitro experiment of β2AR internalization, which showed that β2AR internalization was induced by 10 nmol mL^−1^ formoterol treatment only but not by lower doses of formoterol. Thus, it is likely that β2AR desensitization may have antagonized the protective effect of high‐dose formoterol on EPO‐induced AAA, as supported by our finding that gene knockdown of β2AR blocked the suppressive effect of formoterol on VSMC senescence‐induced by EPO. However, the detailed mechanism requires further investigation.

There were several limitations to this study. First, medium‐dose formoterol protected ApoE^−/−^ mice from AAA only in the EPO‐induced AAA model and it is unknown whether formoterol is equally effective in AngII‐induced AAA model. However, our previous study found that in ApoE^−/−^ mice, EPOR knockdown decreased the incidence of AAA in AngII model, indicating that EPO/EPOR signaling is essential for AngII‐induced AAA.^[^ ^17^ ^]^ Thus, it is likely that formoterol treatment may also benefit AngII‐induced AAA albeit further experiment is needed for confirmation. Second, although our study provided solid evidence that high‐dose formoterol exerted no direct harmful effect on mouse aortic tissues and β2AR internalization is the most likely mechanism for the abolishing effect of high‐dose formoterol on AAA development, further studies are required to elucidate the detailed mechanism.

## 4 Conclusion

**Figure 9:**
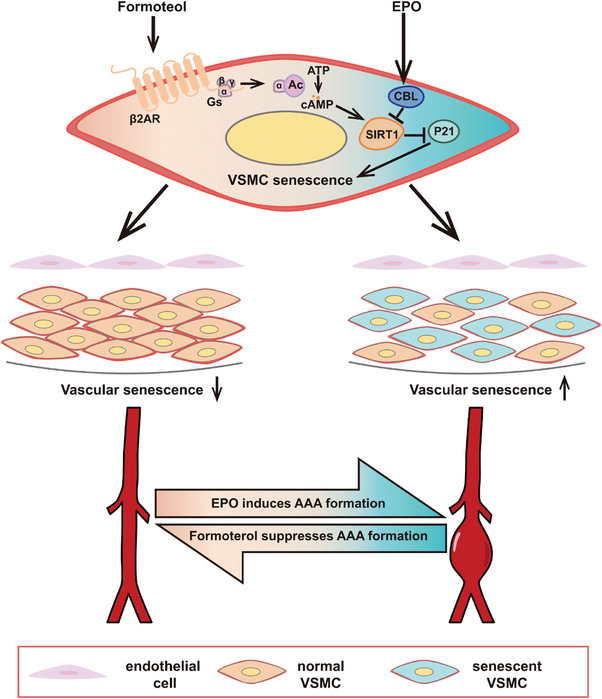
Schematic diagram showing the mechanism of therapeutic effects of medium‐dose formoterol on EPO‐induced AAA. Formoterol binds to β2AR and activates cAMP, which increases SIRT1 protein expression, leading to suppressed VSMC senescence induced by EPO. In contrast, SIRT1 is downregulated by EPO via activation of CBL, resulting in aggravated VSMC senescence. Thus, medium‐dose formoterol attenuated EPO‐induced AAA via β2AR/cAMP/SIRT1 pathways, which provides a promising medication for the treatment of AAA.

With bioinformatic and experimental approaches, we have demonstrated that medium‐dose formoterol treatment attenuated EPO‐induced AAA mainly by targeting VSMC senescence via β2AR/cAMP/SIRT1 signals (**Figure** **9**). However, high‐dose formoterol treatment exerted a null effect on EPO‐induced AAA possibly via β2AR internalization. Thus, medium‐dose formoterol provides a novel medication for the treatment of AAA.

## 5 Experimental Section

### Animal Model

All animal experiments were approved by the ethics committee in Qilu Hospital of Shandong University and were performed under the Animal Management Rules of the Chinese Ministry of Health. One hundred eight‐week‐old male ApoE^−/−^mice were obtained from GemPharmatech Co., Ltd, Nanjing, China. The in vivo experiments consisted of two parts. In the first part of in vivo experiment, 100 eight‐week‐old male ApoE^−/−^mice were obtained from GemPharmatech Co., Ltd, Nanjing, China. All mice were fed on a normal diet for 4 weeks because high‐fat diet feeding was not required in the EPO‐induced AAA model,^[^ ^17^ ^]^ and kept in a 12‐hour light/12‐hour dark cycle with food and water freely available. All mice were randomly divided into 5 groups: vehicle group that received an intraperitoneal injection of 0.2 mL saline once a day for 28 days (*n =* 20), EPO group that received an intraperitoneal injection of 10 000 IU kg^−1^ EPO once a day for 28 days (*n =* 20), EPO+low‐dose formoterol group that received an intraperitoneal injection of 10 000 IU kg^−1^ EPO once a day for 14 days and an intraperitoneal injection of 10 000 IU kg^−1^ EPO plus 0.3 mg kg^−1^ formoterol injection once a day for additional 14 days (*n =* 20), EPO+medium‐dose formoterol group that received an intraperitoneal injection of 10 000 IU kg^−1^ EPO once a day for 14 days and an intraperitoneal injection of 10 000 IU kg^−1^ EPO plus 1 mg kg^−1^ formoterol injection once a day for additional 14 days (*n =* 20), and EPO+high dose formoterol group that received an intraperitoneal injection of 10 000 IU kg^−1^ EPO once a day for 14 days and an intraperitoneal injection of 10 000 IU kg^−1^ EPO plus 3 mg kg^−1^ formoterol injection once a day for additional 14 days (*n =* 20). The three doses of formoterol were selected based on literature recommendations.^[^ ^24^, ^25^ ^]^ A schematic diagram showing the experiment procedure of ApoE^−/−^ mice is given in Figure S2 (Supporting Information).

In the second in vivo experiment, 32 eight‐week‐old male ApoE^−/−^ mice were randomly divided into 4 groups: vehicle group that received an intraperitoneal injection of saline once a day for 28 days, low‐dose formoterol group that received an intraperitoneal injection of saline once a day for 14 days and an intraperitoneal injection of saline plus 0.3 mg kg^−1^ formoterol injection once a day for additional 14 days (*n =* 8), medium‐dose formoterol group that received an intraperitoneal injection of saline once a day for 14 days and an intraperitoneal injection of saline plus 1 mg kg^−1^ formoterol injection once a day for additional 14 days (*n =* 8), high‐dose formoterol group that received an intraperitoneal injection of saline once a day for 14 days and an intraperitoneal injection of saline plus 3 mg kg^−1^ formoterol injection once a day for additional 14 days (*n =* 8). A schematic diagram showing the protocol of this part of the experiments is given in Figure S2 (Supporting Information).

### Cell Experiments

VSMC were washed and incubated in DMEM and then applied to the following six parts of the in vitro experiments. In the first part of the in vitro experiments, to examine the dose‐response relation between formoterol treatment and VSMC senescence, VSMC were divided into 6 groups, which were treated with vehicle (PBS), 5IU mL^−1^ EPO, 5IU mL^−1^ EPO+0.01 nmol mL^−1^ formoterol (S2020, Selleck, USA), 5IU mL^−1^ EPO+0.1 nmol mL^−1^ formoterol, 5IU mL^−1^ EPO+1 nmol mL^−1^ formoterol and 5IU mL^−1^ EPO+10 nmol mL^−1^ formoterol, respectively. As treatment with 5IU mL^−1^ EPO+0.1 nmol mL^−1^ formoterol significantly increased SIRT1 expression and decreased P21 expression, we chose 5IU mL^−1^ EPO+0.1 nmol mL^−1^ formoterol for latter experiments.

In the second part of the in vitro experiments, to explore the role of endogenous β2AR in the inhibitive effect of formoterol on VSMC senescence, VSMC were transfected with specific siRNA against β2AR or negative control. VSMC were divided into 6 groups, which were treated with siNC transfection + vehicle (PBS), siNC transfection + EPO, siNC transfection + EPO+ 0.1 nmol mL^−1^ formoterol group, siβ2AR transfection + vehicle (PBS), siβ2AR transfection + EPO, and siβ2AR transfection + EPO+ 0.1 nmol mL^−1^ formoterol, respectively. Twenty‐four hours after transfection, VSMC were treated with vehicle (PBS), EPO and EPO+ 0.1 nmol mL^−1^ formoterol, respectively, as described above.

In the third part of the in vitro experiments, to unveil the role of cAMP in the mechanism underlying the effects of formoterol, we used the inhibitor of membrane adenylyl cyclase, SQ22535(S8283, Selleck, USA) and dimethyl sulfoxide (DMSO) as solvent control. VSMC were divided into 6 groups, which were treated with control (DMSO)+vehicle (PBS), control (DMSO)+EPO, control (DMSO)+EPO+0.1 nmol mL^−1^ formoterol, SQ22535+PBS, SQ22535+EPO, and SQ22535+EPO+0.1 nmol mL^−1^ formoterol. The dose of SQ22535 was chosen as 80 µM as suggested by previous experiments.^[^ ^13^ ^]^ The cell treatment described in the first to the third part of the in vitro experiments lasted for 24 h before collection according to the previous study.^[^ ^17^ ^]^

In the fourth part of the in vitro experiments, to detect the effect of EPO on CBL/SIRT1 colocalization, VSMC were treated with vehicle (PBS) and 5IU mL^−1^ EPO (287‐TC‐500, R&D Systems, USA), respectively. The cell treatment lasted for 4 h before collection as indicated by a previous study.^[^ ^34^ ^]^

In the fifth part of the in vitro experiments, to explore the role of endogenous CBL in the VSMC senescence induced by EPO, VSMC were transfected with siCBL or siNC. VSMC were divided into 4 groups, which were treated with siNC transfection + vehicle (PBS), siNC transfection + 5IU mL^−1^ EPO, siCBL transfection + vehicle (PBS), and siCBL transfection + 5IU mL^−1^ EPO, respectively. Twenty‐four hours after transfection, VSMC were treated with vehicle (PBS) and 5IU mL^−1^ EPO for 24 h, respectively.

In the sixth part of the in vitro experiments, to explore the effect of different doses of formoterol on β2AR internalization, VSMC were divided into five groups treated with vehicle (PBS), 0.01 nmol mL^−1^ formoterol, 0.1 nmol mL^−1^ formoterol, 1 nmol mL^−1^ formoterol and 10 nmol mL^−1^ formoterol, respectively. The cell treatment lasted for 90 min before collection according to a previous study.^[^ ^47^ ^]^ The entire cellular experiment procedures are illustrated in Figure S6 (Supporting Information).

### Transfection of Cells

The sequence of the siRNA oligos was as follows: mouse CBL sequences: sense 5′‐ UCGGAUUACUAAAGCAGAU‐3′, antisense 5′‐AGCCUAAUGAUUUCGUCUA‐3′; mouse β2AR sequences: sense 5′‐ UUUGUCUAUCUUCUGCAGCTT‐3′, antisense 5′‐GCUGCAGAAGAUAGACAAATT‐3′; mouse SIRT1 sequences: sense 5′ GGAUGAAAGUGAAAUUGAA‐3′, antisense 5′‐CCUACTTTCACUUUAACUU‐3′; negative control (NC) sequences: sense 5′‐UUCUCCGAACGUGUCACGUTT‐3′, antisense 5′‐ ACGUGACACGUUCGGAGAATT‐3′. All siRNAs listed above were obtained from Keyybio, China. The VSMC were transfected with Lipofectamine RNAiMAX (#13 778 150 Thermo Fisher, USA) as described in the supplier's protocols.

### Statistical Analysis

Statistical analysis was performed with GraphPad Prism 8 or R (version 3.6.3). Continuous data were expressed as mean and standard error of the mean (SEM). Categorical data were presented as numbers (%). The significance of the data was tested and detailed in each figure, indicated by *p*‐values. The number of biological and technical repeats in each experimental group was indicated in the corresponding figure legend. Fisher's exact test was applied to the comparisons of AAA incidence and Kaplan‐Meier curves together with the log‐rank (Mantel‐Cox) test was used for survival comparison between groups. To assess Gaussian distribution, ShapiroWilk test was taken for data in each experimental group. Unpaired two‐tailed Student's t‐tests with Welch's correction were applied to determine the statistical difference between two groups with normal distribution. one‐way ANOVA followed by Tukey post hoc tests were performed to determine the statistical difference between multiple groups with one variable and normal distribution. To compare multiple groups with more than one variable, two‐way ANOVA followed by Tukey post hoc tests was used. When a Gaussian distribution cannot be confirmed, the Kruskal‐Wallis test followed by the Nemenyi post hoc test for ≥3 groups was used.

### Ethical Statement

All animal experiments were approved by the Ethic committee in Qilu Hospital of Shandong University (DWLL‐2023‐039) and were performed under the Animal Management Rules of the Chinese Ministry of Health

## Conflict of Interest

The authors declare no conflict of interest.

## Supporting information

Supporting Information

## Data Availability

The data that support the findings of this study are available from the corresponding author upon reasonable request.;
