# Supplementary material for: Medium‐Dose Formoterol Attenuated Abdominal Aortic Aneurysm Induced by EPO via β2AR/cAMP/SIRT1 Pathway
Source: Adv Sci (Weinh). 2024 Feb 14;11(15):2306232. doi: 10.1002/advs.202306232 (PMC11022707; doi:10.1002/advs.202306232)
Supplement: Supplementary file 1 — Supporting Information [file ADVS-11-2306232-s001.pdf]

ADVANCED SCIENCE
Open Access

Supporting Information

for *Adv. Sci.*, DOI 10.1002/advs.202306232

Medium-Dose Formoterol Attenuated Abdominal Aortic Aneurysm Induced by EPO via β2AR/cAMP/SIRT1 Pathway

Jianlin Zhang, Yu Cao, Ruiqing Ren, Wenhai Sui, Yun Zhang, Meng Zhang* and Cheng Zhang**

Supporting Information

Medium-dose formoterol attenuated abdominal aortic aneurysm induced by EPO via β2AR/cAMP/SIRT1 pathway

*Jianlin Zhang, Yu Cao, Ruiqin Ren, Wenhai Sui, Yun Zhang *, Meng Zhang*, and Cheng Zhang**

1.Materials and methods

1.1. Animal model

All animal experiments were approved by the Ethic committee in Qilu Hospital of Shandong University and were performed under the Animal Management Rules of the Chinese Ministry of Health.

The PROC POWER procedure in the SAS software was applied to estimate the sample size of experimental animals. The significance threshold(α) and power(1-β) were set at 0.05 and 80%, respectively. Effect sizes between groups was based on preliminary experiments. The *in vivo* experiments were consisted of two parts. In the first part of *in vivo* experiment, 100 eight-week-old male $ApoE^{-/-}$mice were obtained from GemPharmatech Co.,Ltd, Nanjing, China. All mice were fed on a normal diet for 4 weeks because high-fat diet feeding was not required in our EPO-induced AAA model [17], and kept in 12-hour light/12-hour dark cycle with food and water freely available. All mice were randomly divided into 5 groups: vehicle group that received an intraperitoneal injection of 0.2mL saline once a day for 28 days (*n*=20), EPO group that received an intraperitoneal injection of 10000 IU kg^{-1} EPO once a day for 28 days (*n*=20), EPO+low-dose formoterol group that received an intraperitoneal injection of 10000 IU kg^{-1} EPO once a day for 14 days and an intraperitoneal injection of 10000 IU kg^{-1} EPO plus 0.3mg kg^{-1} formoterol injection once a day for additional 14 days (*n*=20), EPO+medium-dose formoterol group that received an intraperitoneal injection of 10000 IU kg^{-1} EPO once a day for 14 days and an intraperitoneal injection of 10000 IU

kg^{-1} EPO plus 1mg kg^{-1} formoterol injection once a day for additional 14 days (*n*=20), and EPO+high dose formoterol group that received an intraperitoneal injection of 10000 IU kg^{-1} EPO once a day for 14 days and an intraperitoneal injection of 10000 IU kg^{-1} EPO plus 3mg kg^{-1} formoterol injection once a day for additional 14 days (*n*=20). The three doses of formoterol were selected based on literature recommendations [24,25] A schematic diagram showing the experiment procedure of $ApoE^{-/-}$ mice was given in Figure S2.

In the second *in vivo* experiment, 32 eight-week-old male $ApoE^{-/-}$ mice were randomly divided into 4 groups: vehicle group that received an intraperitoneal injection of saline once a day for 28 days, low-dose formoterol group that received an intraperitoneal injection of saline once a day for 14 days and an intraperitoneal injection of saline plus 0.3mg kg^{-1} formoterol injection once a day for additional 14 days (*n*=8), medium-dose formoterol group that received an intraperitoneal injection of saline once a day for 14 days and an intraperitoneal injection of saline plus 1mg kg^{-1} formoterol injection once a day for additional 14 days (*n*=8), high-dose formoterol group that received an intraperitoneal injection of saline once a day for 14 days and an intraperitoneal injection of saline plus 3mg kg^{-1} formoterol injection once a day for additional 14 days (*n*=8). A schematic diagram showing the protocol of this part of experiments was given in Figure S2.

1.2. Measurement of blood pressure

A non-invasive tail-cuff system (Softron BP-98A, Tokyo, Japan) was used to measure heart rate and blood pressure, and all mice were trained first to adapt to the device to ensure reproducible measurements. Heart rate and blood pressure were measured between 9:00 a.m. and noon by the same investigator and the values of heart rate and blood pressure in each mouse were recorded as the mean of three consecutive measurements.

1.3. Serum lipid assay

At the end of the experiment, all mice were fasted for 12 hours and then blood samples were taken from the cardiac apex of each mouse after euthanasia by an intraperitoneal

injection of overdose pentobarbital (80mg kg^{-1}). The serum levels of total cholesterol (TC), triglycerides (TG), low-density lipoprotein cholesterol (LDL-C), and high-density lipoprotein cholesterol (HDL-C) were measured by enzymic assays, using an automatic biochemistry analyzer (Chemray 240, Rayto, Shenzhen, China).

1.4. Histopathological analyses

After dissection of surrounding connective tissue, aortas were photographed with a ruler aside. An investigator blind to mouse grouping measured the outer diameter of the maximal expanded portion of the abdominal aorta in the images with the utility of ImageJ 1.53k software (Wayne Rasband, USA). AAA was defined as the increasement in the outer diameter of the abdominal aorta by no less than 50% relative to the vehicle group (*4*). Then the aortic segment from the ascending aorta to the bifurcation of the common iliac artery were isolated, perfused with 4% PFA for 24h and embedded in paraffin. For characterization of cross sections, the suprarenal region of the abdominal aorta was cut into 5μm-thick sections at an interval of 500 μm and at least 10 sections were analyzed in each mouse.

1.5. Histopathological staining

For immunohistochemical (IHC) staining, the aortic tissues of the maximal expanded portion of the abdominal aortas were cut into 5μm-thick sections. After deparaffinization, rehydration, 10-minute 3%H_2O_2 bath and 1-hour blockage with bovine serum albumin (BSA) at room temperature, the following primary antibodies were added to the slides and incubated at 4°C overnight: MMP2 antibody (ab235167, 1:200 dilution, Abcam, UK), MMP9 antibody (ab283575, 1:200 dilution; Abcam, UK), IL-6 antibody (21865-1-AP, 1:200 dilution; Proteintech, USA), MCP1 antibody (ab214819, 1:200 dilution; Abcam, UK), VCAM antibody (ab134047, 1:200 dilution; Abcam, UK) and α-SMA antibody (ab5694, 1:200 dilution; Abcam, UK). Horseradish peroxidase (HRP)-conjugated goat anti-rabbit IgG (PV-9001, ZSJB-BIO, China) were applied to the sections for 20 minutes at room temperature. The negative controls were displayed with sections reacting with non-immune IgG as well as secondary antibodies. DAB (ZLI-9018, ZSJB-BIO, China) was then added to visualize the positive staining

area. The ratio of positive staining area to the field area was measured by Image-Pro-Plus 6.0 software (Media Cybernetics, USA) by one investigator blind to animal grouping.

For immunofluorescent staining, 5 μm-thick sections derived from the maximally expanded portion of the abdominal aorta were used. After antigen retrieval subsequent to deparaffinization, sections were blocked with 5% BSA for 30 minutes at room temperature and incubated with the following primary antibodies at 4°C overnight: αSMA antibody (ab7817, 1:500 dilution; Abcam, UK) or (ab5694, 1:200 dilution; Abcam, UK), β2AR antibody (ab182136, 1:200 dilution; Abcam, UK), SIRT1 antibody (ab110304, 1:200 dilution; Abcam, UK), γH2AX antibody (phospho S139) (ab2893, 1:200 dilution; Abcam, UK). On the next day, goat anti-mouse IgG H&L (Alexa Fluor® 488; ab150113, 1:200 dilution; Abcam, UK) or goat anti-rabbit IgG H&L (Alexa Fluor® 594; ab150080, 1:200 dilution; Abcam, UK) were applied to the sections and incubated for 1hour at room temperature. The negative controls were displayed with sections reacting with non-immune IgG as well as secondary antibodies. The slides were visualized by an electric upright microscope (DS-Ri2, Nikon, Japan) and the Pearson's R value for co-localization was calculated by ImageJ 1.53k software (Wayne Rasband, USA).

1.6. Zymography

Zymography was performed with an MMP gelatin zymography kit (GenMed Scientific Inc., USA). The protein extracts from the mouse aorta were separated by electrophoresis in SDSPAGE gels containing 0.1% gelatin to measure the activity of MMP2 and MMP9. Gels were washed with renaturing buffer for 1 hour at room temperature and further incubated with developing buffer at 37°C overnight. After incubation, gels were stained with Coomassie Brilliant Blue and then destained with the buffer until clear white bands appeared on the blue background. The activity of MMP2 and MMP9 was calculated by ImageJ 1.53k software (Wayne Rasband, USA).

1.7. TUNEL assay

Cell apoptosis of the aortic tissues was detected by TUNEL using the In Situ Cell Death Detection Kit, (Roche, Germany) following the manufacturer's instructions. 5μm-thick sections derived from the maximal expanded portion of the abdominal aorta were used for TUNEL assay. After deparaffinization, rehydration and permeabilization with 0.1% Triton X-100, the sections were incubated with working solution in 37°C for 1 hour. The working solution was composed of enzyme solution and label solution at the ratio of 1:9. The slides were visualized by an electric upright microscope (DS-Ri2, Nikon, Japan) and the positive rate of each group was calculated by ImageJ 1.53k software (Wayne Rasband, USA).

1.8. Isolation of primary vascular smooth muscle cells

Primary vascular smooth muscle cells (VSMC) were isolated as previously described [7]. In brief, mice aged 6-8 weeks were euthanatized by injection of an overdose pentobarbital (80mg kg^{-1}), and after dissection of surrounding connective tissue, the aortas were immediately transferred to a sterile dish filled with phosphate buffered saline (PBS). The aortic tissues were then cut into pieces and transferred to a sterile dish containing 80% Dulbecco's modified Eagle's medium (DMEM) and 20% fetal bovine serum mixed with 100 μg mL^{-1} streptomycin and 100U mL^{-1} penicillin at 37°C in a humidified atmosphere of 5% CO_2 and 95% air for at least 5 days. Three to six passages of VSMC at 70-80% confluence was used for the *in vitro* experiment.

1.9. Cell experiments

VSMC were washed and incubated in DMEM, and then applied to the following six parts of the *in vitro* experiments. In the first part of the *in vitro* experiments, to examine the dose-response relation between formoterol treatment and VSMC senescence, VSMC were divided into 6 groups, which were treated with vehicle (PBS), 5IU mL^{-1} EPO, 5IU mL^{-1} EPO+0.01 nmol mL^{-1} formoterol (S2020, Selleck, USA), 5IU mL^{-1} EPO+0.1 nmol mL^{-1} formoterol, 5IU mL^{-1} EPO+1 nmol mL^{-1} formoterol and 5IU mL^{-1} EPO+10 nmol mL^{-1} formoterol, respectively. As treatment with 5IU mL^{-1} EPO+0.1 nmol mL^{-1} formoterol significantly increased SIRT1 expression and decreased P21 expression, we chose 5IU mL^{-1} EPO+0.1 nmol mL^{-1} formoterol for latter experiments.

In the second part of the *in vitro* experiments, to explore the role of endogenous β2AR in the inhibitive effect of formoterol on VSMC senescence, VSMC were transfected with specific siRNA against β2AR or negative control. VSMC were divided into 6 groups, which were treated with siNC transfection + vehicle (PBS), siNC transfection + EPO, siNC transfection + EPO+ 0.1 nmol mL^{-1} formoterol group, siβ2AR transfection + vehicle (PBS), siβ2AR transfection + EPO, and siβ2AR transfection + EPO+ 0.1 nmol mL^{-1} formoterol, respectively. Twenty-four hours after transfection, VSMC were treated with vehicle (PBS), EPO and EPO+ 0.1 nmol mL^{-1} formoterol, respectively, as described above.

In the third part of the *in vitro* experiments, to unveil the role of cAMP in the mechanism underlying the effects of formoterol, we used the inhibitor of membrane adenylyl cyclase, SQ22535(S8283, Selleck, USA) and dimethyl sulfoxide (DMSO) as solvent control. VSMC were divided into 6 groups, which were treated with control (DMSO)+vehicle (PBS), control (DMSO)+EPO, control (DMSO)+EPO+0.1 nmol mL^{-1} formoterol, SQ22535+PBS, SQ22535+EPO, and SQ22535+EPO+0.1 nmol mL^{-1} formoterol. The dose of SQ22535 was chosen as 80 μM as suggested by previous experiments (*8*). The cell treatment described in the first to third part of the *in vitro* experiments lasted for 24 hours before collection according to our previous study (*17*).

In the fourth part of the *in vitro* experiments, to detect the effect of EPO on CBL/SIRT1 colocalization, VSMC were treated with vehicle (PBS) and 5IU mL^{-1} EPO (287-TC-500, R&D Systems, USA), respectively. The cell treatment lasted for 4 hours before collection as indicated by a previous study [34].

In the fifth part of the *in vitro* experiments, to explore the role of endogenous CBL in the VSMC senescence induced by EPO, VSMC were transfected with siCBL or siNC. VSMC were divided into 4 groups, which were treated with siNC transfection + vehicle (PBS), siNC transfection + 5IU mL^{-1} EPO, siCBL transfection + vehicle (PBS) and siCBL transfection + 5IU mL^{-1} EPO, respectively. Twenty-four hours after transfection, VSMC were treated with vehicle (PBS) and 5IU mL^{-1} EPO for 24 hours, respectively.

In the sixth part of the *in vitro* experiments, to explore the effect of different doses of formoterol on β2AR internalization, VSMC were divided into 5 groups treated with vehicle (PBS), 0.01 nmol mL^{-1} formoterol, 0.1 nmol mL^{-1} formoterol, 1 nmol mL^{-1} formoterol and 10 nmol mL^{-1} formoterol, respectively. The cell treatment lasted for 90 minutes before collection according to a previous study [47]. The entire cellular experiment procedures were illustrated in Figure S6.

1.9. Transfection of cells

The sequence of the siRNA oligos was as follows: mouse CBL sequences: sense 5'-UCGGAUUACUAAAGCAGAU-3', antisense 5'-AGCCUAAUGAUUUCGUCUA-3'; mouse β2AR sequences: sense 5'- UUUGUCUAUCUUCUGCAGCTT-3', antisense 5'-GCUGCAGAAGAUAGACAAATT-3'; mouse SIRT1 sequences: sense 5' GGAUGAAAGUGAAAUUGAA-3', antisense 5'-CCUACTTTCACUUUAACUU-3'; negative control (NC) sequences: sense 5'-UUCUCCGAACGUGUCACGUTT-3', antisense 5'- ACGUGACACGUUCGGAGAATT-3'. All siRNAs listed above were obtained from Keyybio, China. The VSMC were transfected with Lipofectamine RNAiMAX (#13778150 Thermo Fisher, USA) as described in supplier's protocols.

1.10. Immunofluorescence

VSMC were seeded in 12-well dishes with a cover glass inside. After treatment, cells were washed with PBS and fixed with 4% PFA for 15 minutes at room temperature. Then the cells were blocked with 5% BSA for 1 hour at room temperature and incubated with the SIRT1 antibody (ab110304, 1:200 dilution; Abcam, UK), αSMA antibody (ab5694, 1:200 dilution; Abcam, UK), CBL antibody (sc-1651, 1:200 dilution; Santa Cruz, USA) and β2AR antibody (ab182136, 1:200 dilution; Abcam, UK) at 4°C overnight. On the next day, goat anti-mouse IgG H&L (Alexa Fluor® 488) (ab150113, 1:200 dilution; Abcam, UK) and goat anti-rabbit IgG H&L (Alexa Fluor® 594; ab150080, 1:200 dilution; Abcam, UK) were applied to cells for 1 hour at room temperature. An upright electric fluorescence microscope (DS-Ri2, Nikon, Japan) was applied to visualize the cells. The results were analyzed by ImageJ 1.53k software (Wayne Rasband, USA).

1.11. SA-β-gal activity assay

Cells were stained to measure SA-β-gal activity by a commercial kit (C0602, Beyotime, China) according to the manufacturer’s protocols. Briefly, cells seeded on the cover glass in 12-well dishes were washed and fixed with 4% PFA for 15 minutes at room temperature. Next, the cells were incubated at 37°C overnight in darkness with staining solution containing 0.05 mg mL^{-1} 5-bromo-4-chloro-3-indolyl-b-d-galactopyranoside (X-gal). The blue-colored cells were regarded as SA-β-gal positive. The cells were also counterstained with DAPI for nucleus and the SA-β-gal activity-positive cells were detected by light and fluorescence microscopy (DS-Ri2, Nikon, Japan). The results were analyzed by ImageJ 1.53k software (Wayne Rasband, USA).

1.12. Measurement of intracellular cAMP level

Intracellular cAMP level was measured using enzyme-linked immunosorbent assay for quantitative detection of mouse-cAMP kits (TAE-239m, Anoric, China) according to the manufacturer’s instructions.

1.13. Western blot analysis

Protein lysates were extracted from VSMC on ice using RIPA lysis buffer (CW2333, CWBIO, China) supplemented with protease inhibitor cocktail (CW2200, CWBIO, China). Protein lysates of cell membranes were extracted from VSMC using membrane protein extraction kit (PK10015, Proteintech, China). Protein lysates of the whole aortas were extracted by the TRIzol Reagent (Invitrogen, Carlsbad, CA) following manufacturer's instructions. The proteins were separated by SDS-PAGE, transferred to polyvinylidene fluoride (PVDF) membrane, blocked by 5% non-fat milk for 1 hour at room temperature and incubated with the following primary antibodies at 4°C overnight: IL-6 antibody (21865-1-AP, Proteintech, China), MCP1 antibody (ab214819, Abcam, UK), VCAM antibody (ab134047, Abcam, UK), MMP2 antibody (ab235167, , Abcam, UK), MMP9 antibody (ab283575, Abcam, UK), αSMA antibody (ab7817, Abcam, UK), BCL2 antibody (ab124734, Abcam, UK), BAX antibody (ab32503, Abcam, UK), SIRT1 antibody (ab110304, Abcam, UK), P21 antibody (10355-1-AP, Proteintech, China), β2AR antibody (ab182136, Abcam, UK), ATP1A1 antibody

(14418-1-AP, Proteintech, China) and β-actin antibody (#4970, Cell Signaling Technology, USA). On the next day, the membranes were incubated with HRP-conjugated affinipure goat anti-rabbit IgG(H+L) (SA00001-2, Proteintech, China) or HRP-conjugated affinipure goat anti-mouse IgG(H+L) (SA00001-1, Proteintech, China) at room temperature for 1hour. Signals were detected by applying immobilon ECL ultra western HRP substrate (WBULS0500, Millipore, USA) and imaged by luminescent image analyzer (Amersham Imager 800, GE, USA).

2. Computational pipeline

2.1. Data acquisition and differentially expressed genes (DEGs) analysis

The mRNA expression profiling dataset GSE174556 was downloaded from the GEO database. The samples consisted of the entire aortas isolated from ApoE$^{-/-}$ mice receiving either 5000IU kg^{-1} EPO (n=3) or vehicle (n=3) treatment. The dataset was sequenced on the Illumina HiSeq 4000 (Mus musculus; Illumina Inc., USA). All differential expression genes (DEGs) were screened with the "edgeR" package of R software 3.6.3 for background correction and normalization. The normalization and log2 transformation of gene expression was also conducted with "edgeR" package in R. The genes with false discovery rate (FDR) < 0.05 and $|\log2(\text{Fold change})| > 1$ were regarded as statistically significant DEGs.

2.2. Pathway enrichment analysis

The total DEGs as well as the upregulated and downregulated DEGs were enriched with both Gene Ontology (GO) analysis and Kyoto Encyclopedia of Genes and Genomes (KEGG) analysis. The enrichment analyses were performed on Metascape [48] and KOBAS [49]. Results obtained from Metascape were visualized with Cytoscape 3.9.0.

2.3. Screening of potential targeted drugs against EPO-induced AAA

Connectivity Map (CMap) provides a systematic approach for discovering associations among therapeutic agents, gene expression changes, and biological pathways based on genome wide expression profiling to repurpose the "old drugs" for new use (https://clue.io/data) [22,23]. The mechanism of CMap is to find compounds affecting

similar or opposite physiological processes and gene expression profiles by comparison. We submitted (1) the intersection genes between total DEGs and AAA-centered genes including 37 upregulated genes and 87 downregulated genes, (2) 37 upregulated genes and (3) 87 downregulated genes, respectively to CMap in order to find compounds targeting all DEGs and specifically targeting upregulated/downregulated genes. A threshold of (-log10 FDR) > 15 was set to screen potential drugs in the submission of total and upregulated genes. Given the scarcity of drugs found in the submission of the 87 downregulated genes, no candidate reached the standard. Therefore, (-log10 FDR) > 2 was set in this group.

3. Statistical analysis

Statistical analysis was performed with Graphpad Prism 8 or R (version 3.6.3). Continuous data were expressed as mean and standard error of the mean (SEM). Categorical data were presented as number (%). Significance of the data were tested and detailed in each figure, indicated by *P* values. The number of biological and technical repeats in each experimental group was indicated in correspondent figure legend. Fisher's exact test was applied to the comparisons of AAA incidence and Kaplan-Meier curves together with log-rank (Mantel-Cox) test was used for survival comparison between groups. To assess Gaussian distribution, Shapiro-Wilk test was taken for data in each experimental group. And unpaired two-tailed Student's t tests with Welch's correction were applied to determine the statistical difference between two groups with normal distribution. one-way ANOVA followed by Tukey post hoc tests were performed to determine the statistical difference between multiple groups with one variable and normally distribution. To compare multiple groups with more than one variable, two-way ANOVA followed by Tukey post hoc tests was used. When a Gaussian distribution cannot be confirmed, the Kruskal-Wallis test followed by Nemenyi post hoc test for ≥3 groups were used.

Supplemental figures

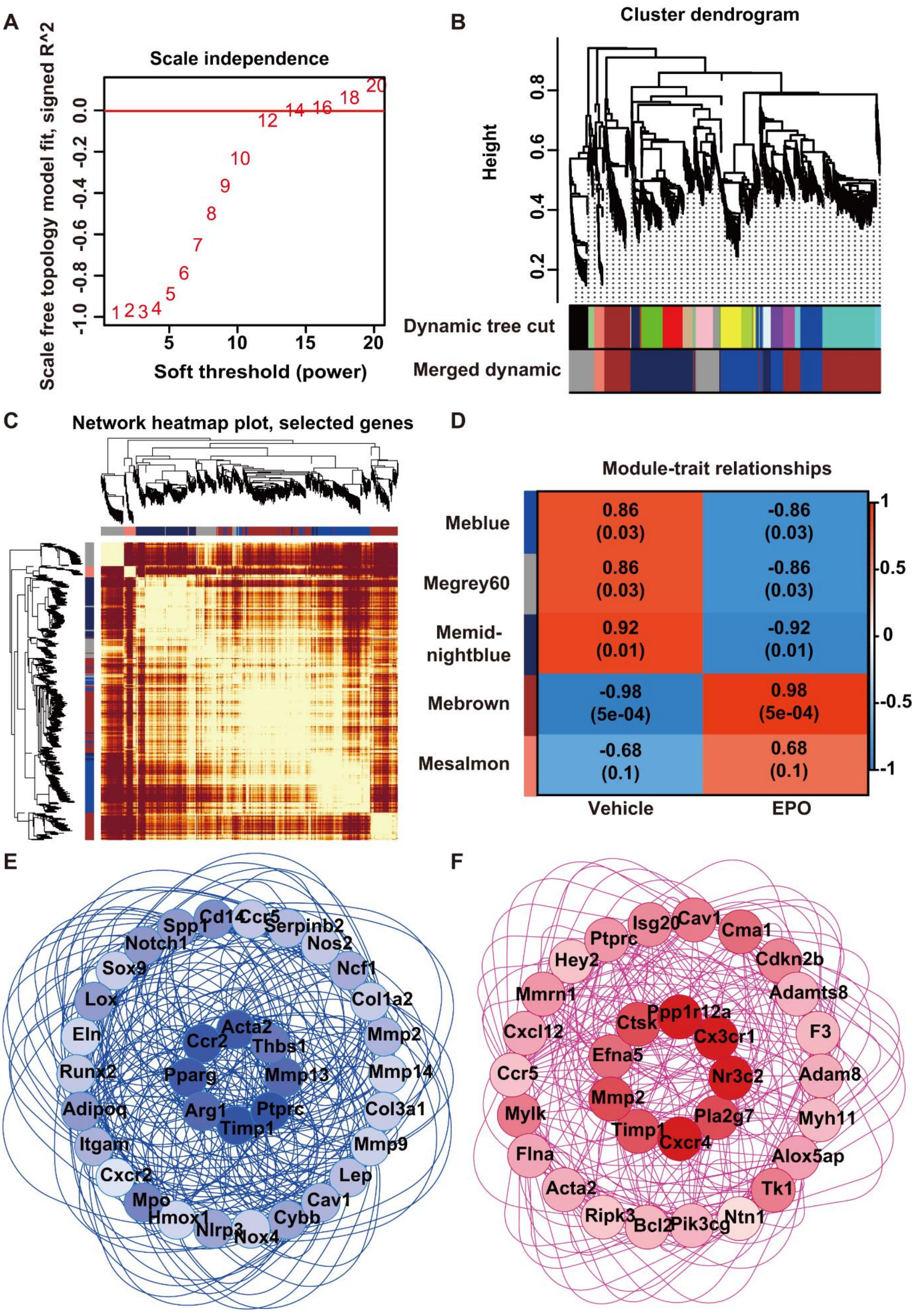
A
Scale independence
Scale free topology model fit, signed R^2
Soft threshold (power)
B
Cluster dendrogram
Height
Dynamic tree cut
Merged dynamic
C
Network heatmap plot, selected genes
D
Module-trait relationships
Meblue
Megrey60
Memid-nightblue
Mebrown
Mesalmon
Vehicle
EPO
0.86 (0.03)
-0.86 (0.03)
0.86 (0.03)
-0.86 (0.03)
0.92 (0.01)
-0.92 (0.01)
-0.98 (5e-04)
0.98 (5e-04)
-0.68 (0.1)
0.68 (0.1)
E
F

Figure S1. Weighted correlation network analysis (WGCNA). A) Detection of outlier in sample clustering. The clusters covered all samples and soft threshold power analysis helped to obtain the scale-free-fit index of the network topology (soft threshold power=14). B) Cluster dendrogram of genes based on co-expression relations determined by WGCNA. C) Heatmap of the topological overlap matrix (TOM) of selected genes. The higher degree of overlap, the darker. D) Module-trait relationships of different modules and disease status. E) Co-expression network of genes in the intersection. F) Protein-protein interaction network of genes in the intersection.

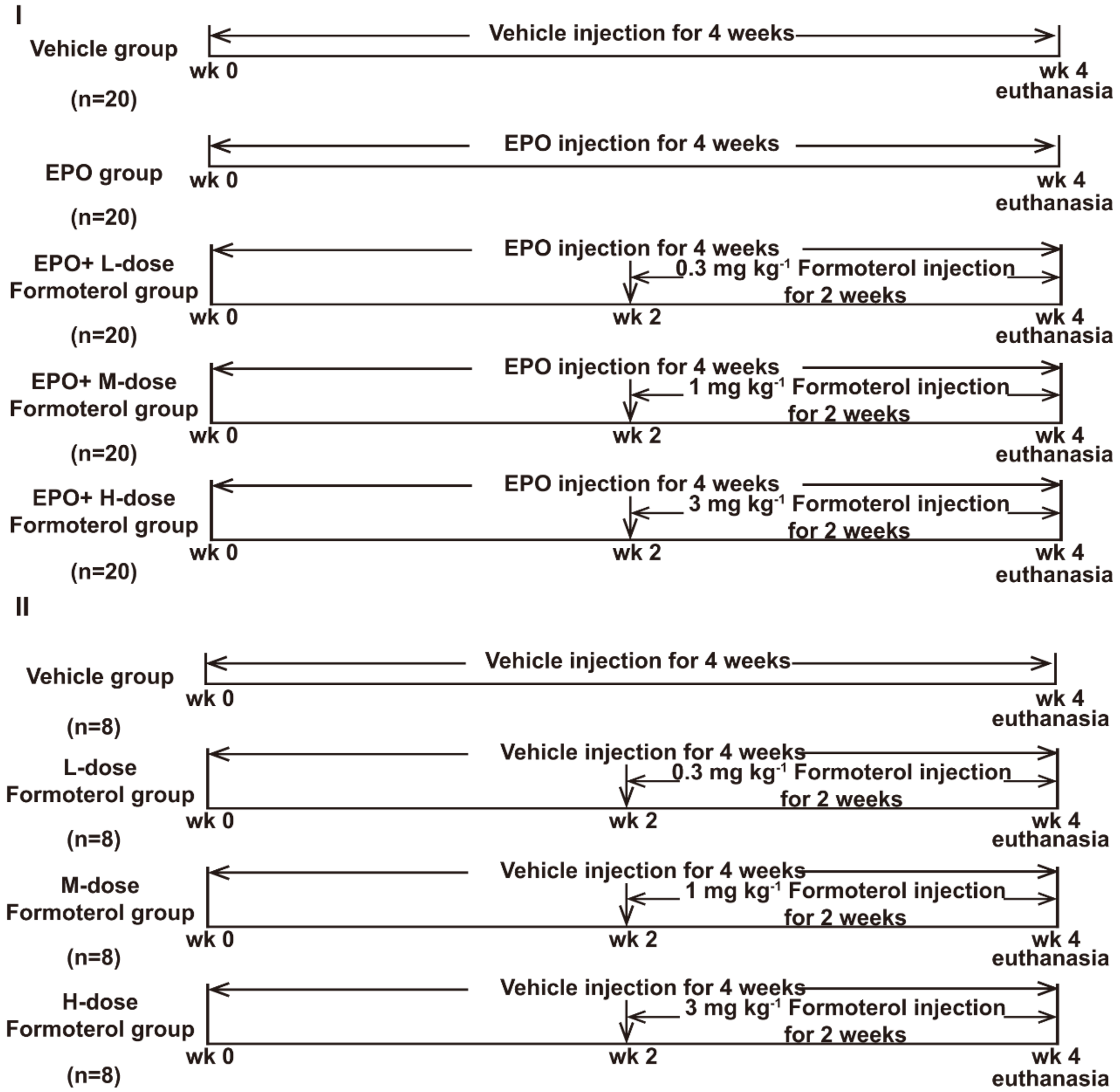

Figure S2. Schematic diagram showing the *in vivo* experiment protocol in ApoE$^{-/-}$ mice.

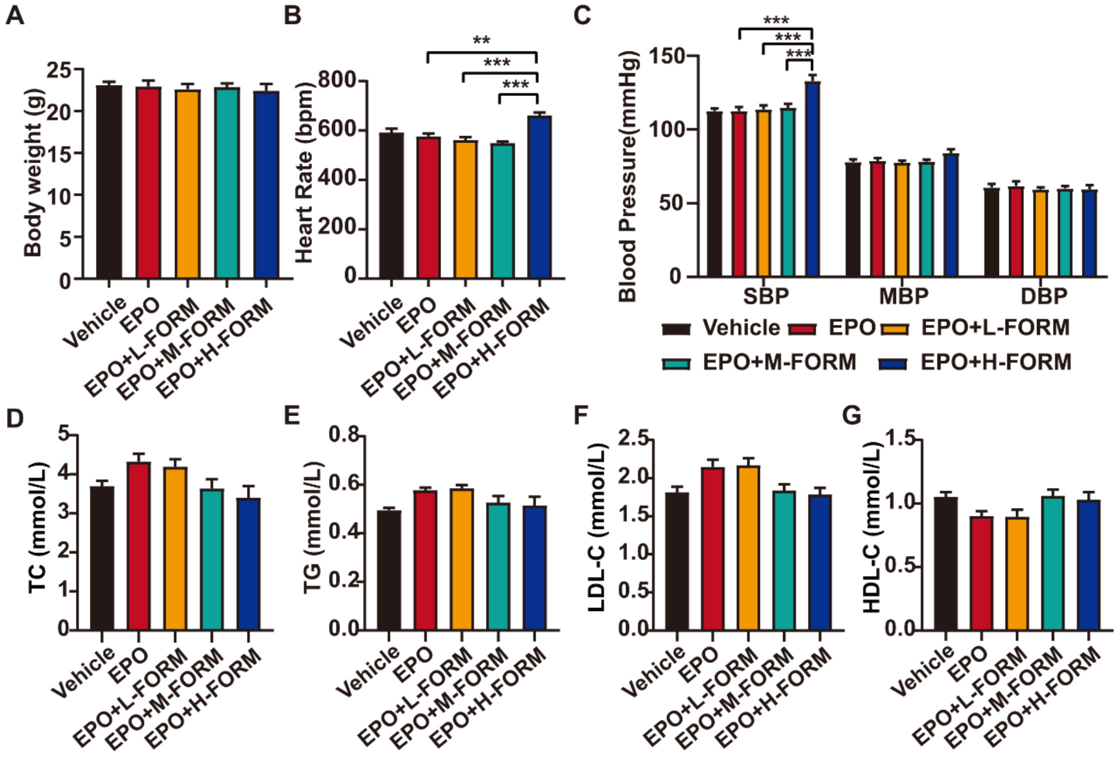

Figure S3. Characteristics and biochemical measurements in five groups of ApoE$^{-/-}$ mice. A) Body weight in five groups of ApoE$^{-/-}$ mice ($n \geq 11$ per group). B) Heart rate in five groups of ApoE$^{-/-}$ mice ($n \geq 11$ per group) C) Systolic, mean and diastolic blood pressure in five groups of ApoE$^{-/-}$ mice ($n \geq 11$ per group) D-G) Serum concentrations of TC, TG, LDL-C and HDL-C in five groups of ApoE$^{-/-}$ mice ($n=7$ per group). $^{**}P<0.01$, $^{***}P<0.001$, one-way ANOVA followed by Tukey test for post hoc comparison, mean ± SEM.

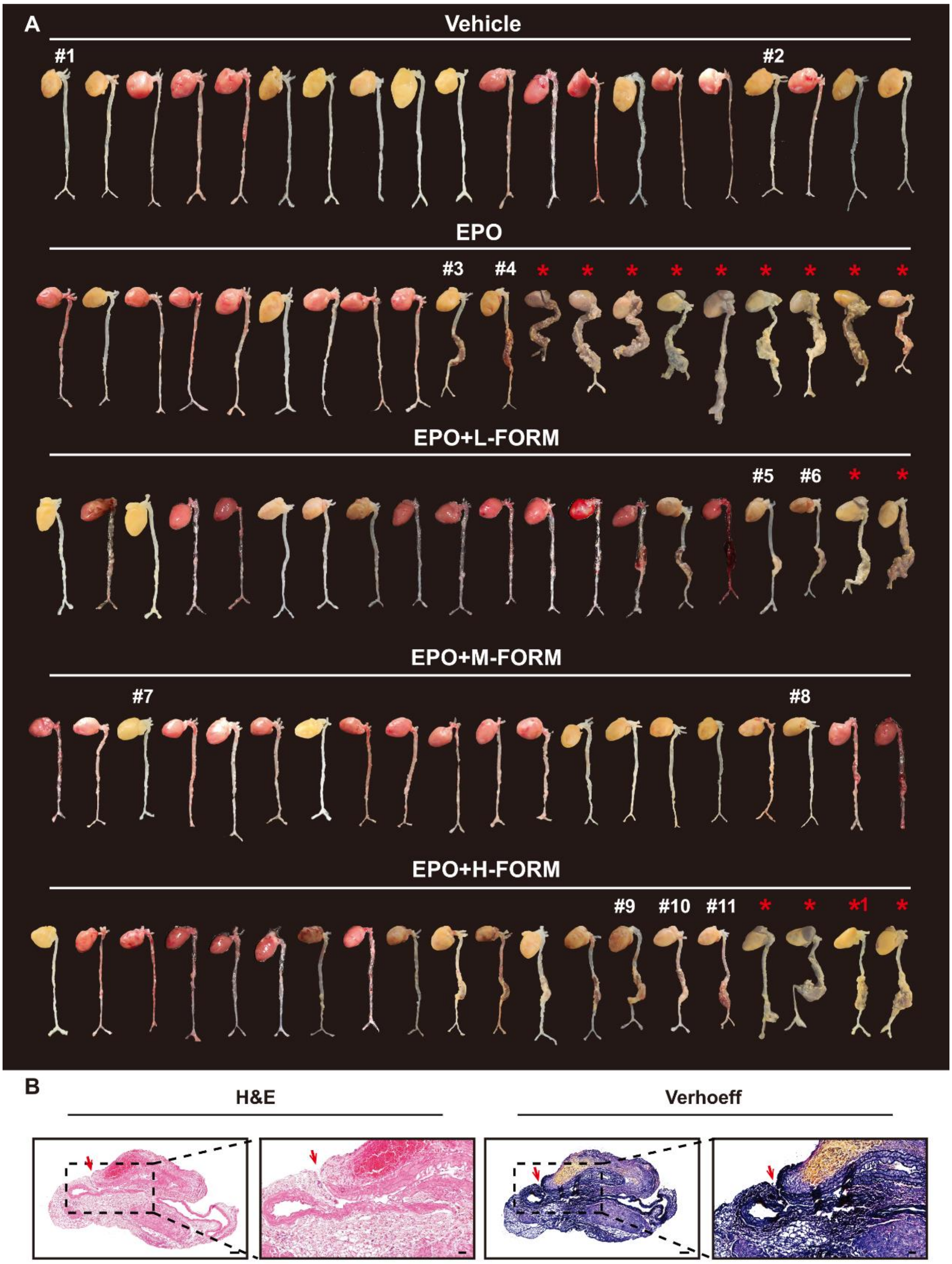

Figure S4. Photographs of aortic specimens in individual ApoE$^{-/-}$ mice. A) Photographs of all aortic specimens in ApoE$^{-/-}$ mice receiving vehicle, EPO, EPO+low-dose formoterol (FORM), EPO+medium-dose FORM and EPO+high-dose FORM treatment, respectively. The pound sign (#) indicates the specimen used in figures. The pound sign

(*) indicates the specimens from the autopsy of mice who died during the experiment. #1 was used for figure 2C,2G,3A,4A 4E and 6A, #2 for figure 2C,5C and 5E #3 for figure 2C, 3A and 4E, #4 for figure 2C, 2G, 4A,5C and 5E, #5 for figure 2C, 2G, 3A,4A and 4E, #6 for figure 2C, #7, for figure 2C, 2G, 5C and 5E, #8 for figure 2C, 3A, 4A and 4E, #9, for figure 2C and 4E, #10, for figure 3A, and #11 for figure 2C and figure 4A. *1 was used for figure 2G and figure S4B. B) Representative H&E and Verhoeff staining of abdominal aortic section in ApoE$^{-/-}$ mice who died of AAA, showing the ruptured aneurysmal wall as indicated by an arrow. Low (×10, scale bars=25μm) and medium (×20, scale bars=10μm) magnifications were shown. The specimen used in (B) was indicated by *1.

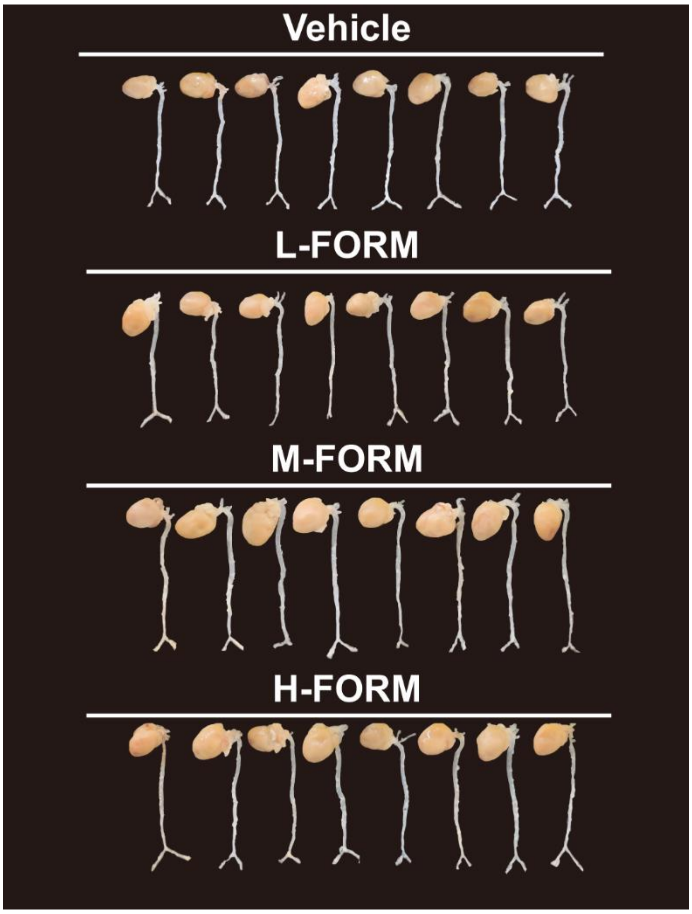

Figure S5. Photographs of all aortic specimens in ApoE$^{-/-}$ mice receiving vehicle, low-dose FORM, medium-dose FORM and high-dose FORM treatment, respectively.

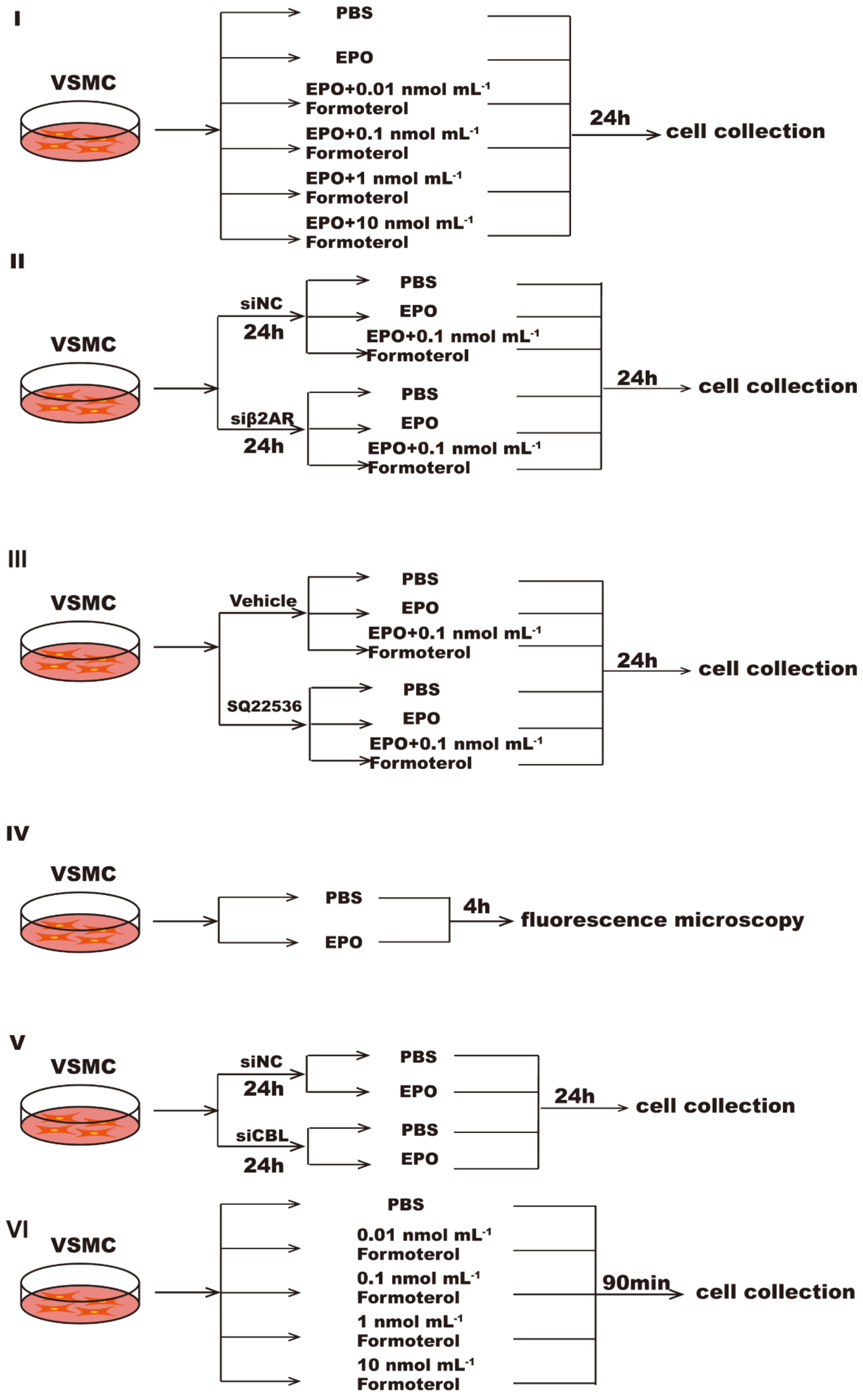

Figure S6. Schematic diagram showing the *in vitro* experimental protocol in VSMC.

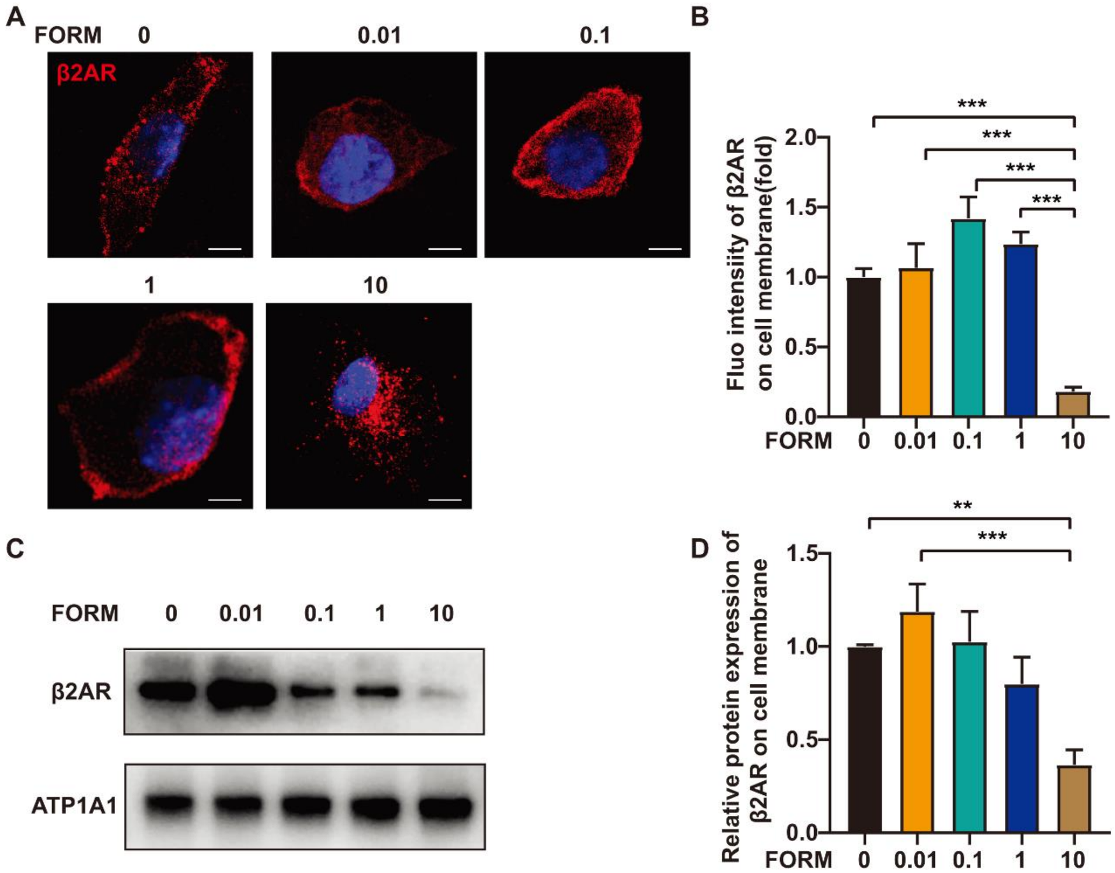

Figure S7. High-dose formoterol (FORM) induced β2AR internalization in VSMC. A) Representative immunofluorescent analysis of β2AR in VSMC receiving vehicle (PBS), 0.01 nmol mL^{-1} FORM, 0.1 nmol mL^{-1} FORM, 1 nmol mL^{-1} FORM and 10 nmol mL^{-1} FORM, respectively. B) Quantitative analysis of fluorescent intensity of β2AR on cell membrane in (A) (n=6 per group). C) Representative western blot analysis of protein expression of β2AR in membrane proteins of VSMC receiving vehicle (PBS), 0.01 nmol mL^{-1} FORM, 0.1 nmol mL^{-1} FORM, 1 nmol mL^{-1} FORM and 10 nmol mL^{-1} FORM, respectively. D) Quantitative analysis of western blot in (C) (n=6 per group). ** P<0.01, *** P<0.001, One-way ANOVA followed by Tukey test for post hoc comparison, mean ± SEM.

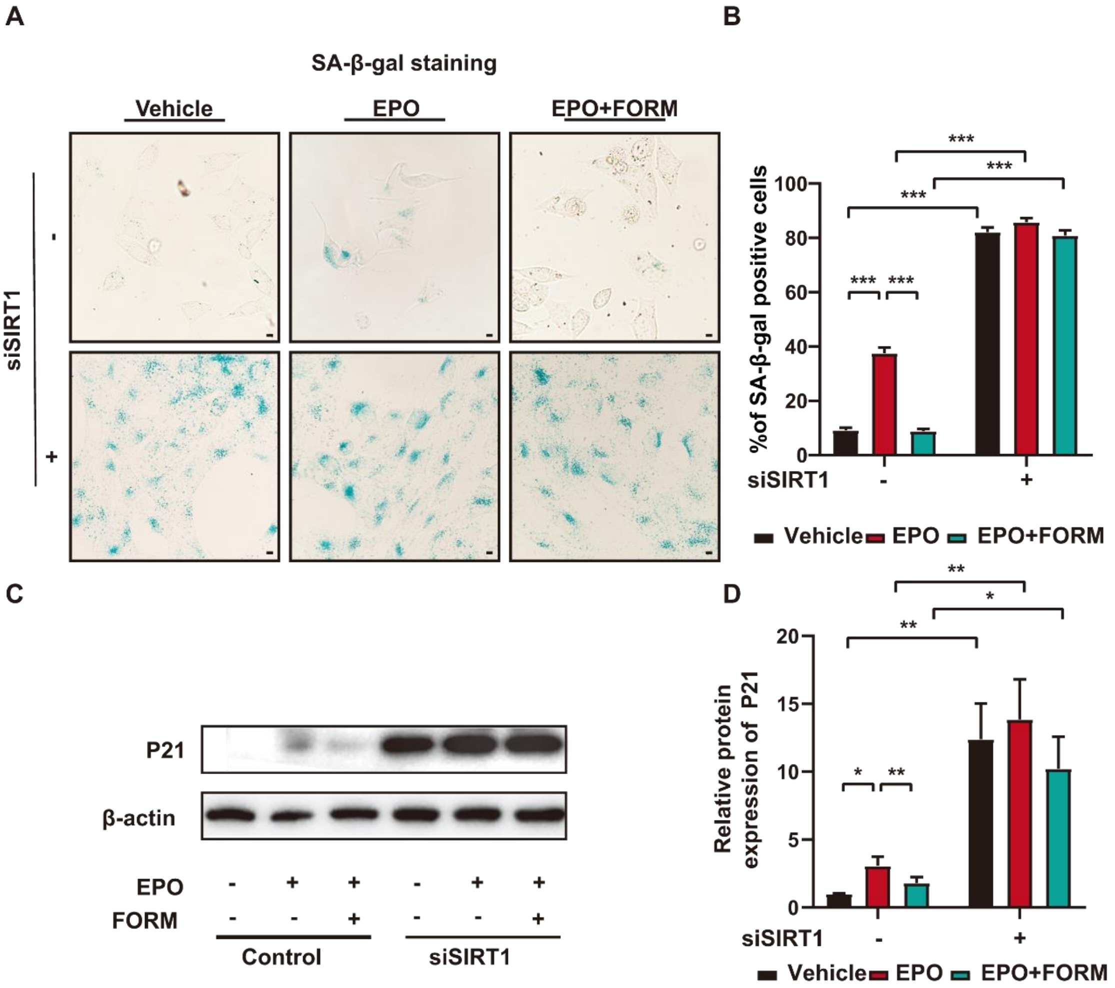

Figure S8. SIRT1 was essential for the effect of formoterol (FORM) on VSMC senescence. A) Representative sections of SA-β-gal staining of VSMC transfected with SIRT1 siRNA (siSIRT1) or negative control siRNA (siNC) and treated with vehicle, EPO and EPO+0.1 nmol mL^{-1} formoterol, respectively (scale bars=10μm). B) Quantitative analysis of percentage of VSMC with positive SA-β-gal staining in (A) (n=6 per group). C) Representative western blot analysis of protein expression of P21 in VSMC transfected with siSIRT1 or siNC and treated with vehicle, EPO and EPO+ 0.1 nmol mL^{-1}formoterol, respectively. D) Quantitative analysis of western blot in (C) (n=6 per group). *$P < 0.05$, ** $P<0.01$, *** $P<0.001$, Two-way ANOVA followed by Tukey test for post hoc comparison, mean ± SEM.
